# An updated systematic review with meta-analysis and meta-regression of the factors associated with human visceral leishmaniasis in the Americas

**DOI:** 10.1186/s40249-025-01274-z

**Published:** 2025-01-30

**Authors:** Anna Gabryela Sousa Duarte, Guilherme Loureiro Werneck, Sarah de Farias Lelis, Thays Santos Mendonça, Daniela Dias Vasconcelos, Tiago Silveira Gontijo, Álisson Oliveira dos Santos, Lucas Edel Donato, Vinícius Silva Belo

**Affiliations:** 1 Universidade Federal de São João del Rei (UFSJ), Campus Centro-Oeste Dona Lindu, Avenida Sebastião Gonçalves Coelho 400, Chanadour, Divinópolis, MG Brazil; 2 Prefeitura Municipal de Divinópolis-Minas Gerais, Secretaria Municipal de Saúde, Divinópolis, MG Brazil; 3 Departamento de Epidemiologia, Universidade Do Estado Do Rio de Janeiro, Rio de Janeiro, RJ Brazil; 4 Ministério da Saúde Do Brasil, Secretaria de Vigilância Em Saúde E Ambiente, Brasília, DF Brazil; 5 Campus de Três Lagoas (CPTL), Universidade Federal de Mato Grosso Do Sul (UFMS), Três Lagoas, MS Brazil

**Keywords:** Leishmaniasis, Epidemiology, Risk factor, Systematic review, Quality data reporting

## Abstract

**Background:**

Human visceral leishmaniasis (VL) is a systemic disease with high case-fatality rates and a widespread distribution. Continuous evaluation of the risk factors for VL is essential to ensure the effective implementation of prevention and control measures. The present study reviews the factors associated with VL in the Americas.

**Methods:**

This systematic review updates a previous 2013 report by including cross-sectional, cohort and case-control studies published between July 2011 and April 2024. Associations between VL and risk factors were analyzed using random-effects meta-analysis, subgroup analysis, and meta-regression models. Studies were classified according to level of evidence using the GRADE approach and the evolution in the quality of investigations was assessed.

**Results:**

Forty-six studies were included in the review and 21 variables were evaluated in the meta-analyses. Combination of all study types revealed that men had greater chances of VL than women, but the association was strong and significant only in case-control studies. Although higher chances of VL in children and in households with dogs or chickens/other fowl were identified in case-control studies, an inverse association was observed in cross-sectional and cohort studies. Higher chances of VL were associated with poor economic/living conditions, individuals living in domiciles with backyards or with seropositive dogs, and individuals with prior contact with infected household members/relatives/neighbors. The level of evidence for associations of VL with sex and age was classified as moderate whilst that for all other associations was either low or very low. The methodological quality of recent studies showed a positive progression but shortcomings were still evident regarding selection criteria and methods of data analysis.

**Conclusion:**

While there is a higher incidence of symptomatic VL among men and children, the likelihood of infection is similar between the groups. There is insufficient evidence to support the claim that the presence of dogs or fowl at the domicile increases the chances of VL. However, socioeconomic and living conditions, as well as previous occurrence of human and canine VL, are influential factors. Future research should be conducted with greater statistical power and using molecular diagnostic techniques, preferably involving cohort studies in diverse Latin American countries.

**Graphical Abstract:**

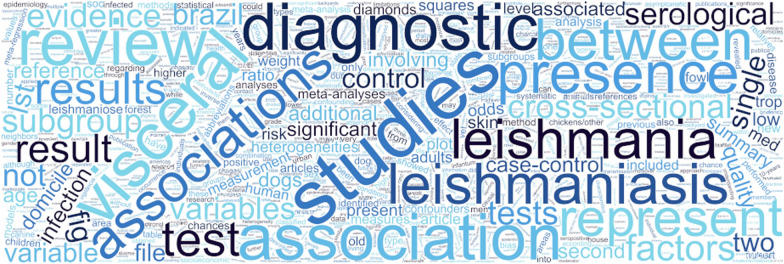

**Supplementary Information:**

The online version contains supplementary material available at 10.1186/s40249-025-01274-z.

## Background

Visceral leishmaniasis (VL) is a systemic disease with a potentially serious course, high fatality rates and a widespread global distribution [1, 2]. The disease has been reported in 13 countries of the Americas with approximately 2000 cases recorded every year over the last decade, the vast majority (90%) of which occurred in Brazil [3].

In urban environments of Latin America, the etiological agent of VL is the protozoan parasite *Leishmania infantum,* the main reservoir of which is generally considered to be the domestic dog (*Canis lupus familiaris*) [4]. Transmission of the parasite to humans occurs mainly through bites of female sandflies of the species *Lutzomyia longipalpis*. Strategies for the prevention and control of the disease target primarily either the vector through the application of residual insecticides, or dogs by conducting epidemiological surveys, protecting animals with insect repellent collars, and euthanasia of seropositive animals [4–7].

The effectiveness of VL prevention and control strategies may vary depending on local epidemiological contexts and the underlying factors driving transmission. Thus, the identification of risk factors is essential to understanding VL determinants and establishing effective intervention programs [8–11]. Furthermore, a comprehensive understanding of the diverse variables associated with the disease, such as age, gender, socioeconomic status, presence of domestic animals, and environmental conditions, is fundamental to the successful reduction or elimination of VL, as these goals can only be achieved through integrated measures [12].

In 2013, a systematic review of studies covering the period between 1980 and June 2011 was published [13], in which the factors associated with human infection by *L. infantum* in the Americas were summarized and the strengths and directions of associations between VL and several explanatory variables were identified. That review revealed that the results of some studies were susceptible to bias and the methodological quality of the investigations required improvement. Since the publication of that review, new research on the topic has been published [14–17] and it is, therefore, opportune to assess the methods and results described in recent primary investigations and to compare them with those described in earlier studies. Such timely review will provide a more consistent rationale regarding the variables already studied and afford new insights into other variables that are still underexplored. Furthermore, examination of a larger number of studies produced over a longer period, taken together with the development of more advanced analytical procedures and improved methodological quality, should enhance the level of evidence and the statistical powers of meta-analyses and open up the possibility of performing more appropriate evaluations of heterogeneity.

In light of the above, the present study updates the 2013 review by including primary research articles on VL published over the last 12 years. In addition, by adopting additional statistical methods, we present novel analyses of the level of evidence for associations between VL and potential risk factors.

## Methods

### Type of study and inclusion and exclusion criteria

The present systematic review with meta-analysis updates the 2013 paper [13] by considering the results derived from epidemiological studies with outcomes measured at an individual level and published between July 2011 and April 2024. The review was elaborated following the Meta-analysis of Observational Studies in Epidemiology (MOOSE) [18] and Preferred Reporting Items for Systematic Reviews and Meta-Analyses 2020 (PRISMA) [19] guidelines, and has been registered on the PROSPERO platform under protocol CRD42020197056.

The selection of original primary studies encompassed all cross-sectional, cohort and case-control studies describing associations between any outcome related to human *L. infantum* infection—prevalence, incidence or identification of clinical cases —, and socioeconomic, environmental, family, and individual variables. No restrictions were applied regarding diagnostic methods.

The exclusion criteria included studies or variables that were purely descriptive or for which the full text or supplementary information requested from the authors could not be obtained, despite direct attempts to contact them via email. A follow-up email was sent, with a response requested within 30 days. Furthermore, studies focusing on variables related to control measures (as the objective of this review was not to evaluate interventions), individual genetic characteristics (as these are non-modifiable factors and outside the scope of this review), or duration of residence in a specific location (as this is a context-specific variable with limited generalizability) were not included.

### Searches, selection of primary studies and extraction of information

Searches of the literature were conducted independently by two of our research team (AGSD and SFL) using the data sources and terms presented in the Additional file 1. No language restrictions were applied for the inclusion of articles. The titles and abstracts of all articles retrieved in the searches were analyzed and those that were not within the scope of the proposed criteria were excluded. After removing duplicates, the remaining articles were read in full to determine whether they would be included in the review. At all stages, cases of disagreement between the two researchers were resolved by consensus with a third researcher (VSB). A standardized Excel spreadsheet was used to extract the following information from individual studies: author(s), year of publication, location, study type, study duration, sample size, design details, exposure variables, number of exposed and unexposed individuals, measures of association or values necessary to calculate them, confidence intervals, *P*-values, direction of associations, outcomes, diagnostic techniques, data analysis methods, and whether confounding factors were controlled.

### Analysis of susceptibility to bias

The limitations and susceptibility to bias of the selected studies were analyzed using the tools described in our previous publication: Strengthening the reporting of observational studies in epidemiology statement and Newcastle–Ottawa Quality Assessment Scale [13]. In addition, the Joanna Briggs Institute appraisal tools were employed to examine the extent to which the primary studies included in the earlier and the present review had addressed the likelihood of bias, with the quality of studies being classified as high, moderate or low [20] (Additional file 2). The scores and classifications attributed to the two groups of studies were compared by means of descriptive statistics.

### Statistical analysis and meta-analytic methods

The statistical combination of association measures from the primary studies took into consideration the similarity between the issues investigated and the need for the association to have been evaluated in at least three studies. The combination of association measures was performed using random effects models and the results were described in terms of odds ratio (*OR*) and confidence interval (*CI*).

The *I*^*2*^ statistic was calculated to determine the proportion of the observed variance between the association measures that represented the true dispersion in effect sizes. Subgroup analyses were performed for risk factors that were addressed in a sufficient number of studies in order to identify variables that might explain heterogeneities in the strength of the associations. The following variables were considered possible moderators of the relationship between predictors and human *L. infantum* infection outcomes and submitted to subgroup analyses: (i) study type: cross-sectional, cohort, case-control; (ii) diagnostic method (method for measuring the outcome): *Leishmania* skin test (LST), serological test, LST plus serological test, other tests, clinical case in case-control studies; (iii) control of confounders: yes, no; (iv) age group: adults, children; and (v) study period: 2013 review, present review. The results of such analyses are shown only for subgroups that adequately explained the heterogeneities, determined by higher heterogeneity between subgroups than within subgroups (indicated by *I*^2^ values), differences in the measures of association between subgroups, or statistical significance in the Q test (*P* < 0.05). However, when stratifying to control for confounders, results were shown for all associations where analysis was possible, regardless of this subgroup’s explanatory capacity. For all variables in which subgroups adequately explained heterogeneities, the association measures were combined only within the groups without obtaining a global measure. This approach was adopted to account for the heterogeneity across subgroups, ensuring that the results accurately represent the distinct characteristics of each subgroup.

In order to determine the potential sources of heterogeneity, random-effects meta-regression analyses were performed, a novel approach not applied in the previous review. We explored heterogeneities in the associations of VL with the variables sex, age, domicile with dogs, and domicile with chickens/other fowl because each variable was addressed in at least 10 primary studies [21]. Moderators that had the potential to explain substantial heterogeneities were selected from subgroup analyses conducted previously. Consequently, the moderators ‘study period’ and ‘age group’ were excluded from the meta-regression models. The moderators ‘study type’, ‘diagnostic method’, and ‘control of confounders’ were initially analyzed using crude meta-regression models, and those with *P* < 0.10 were included in multiple meta-regression models, adopting a similar level of significance [22]. Study type and diagnostic method were not analyzed together by multiple regression models because of the presence of collinearity [assessed using Variance Inflation Factors (VIF), with VIF > 5 indicating collinearity] and, therefore, only the moderator with the highest analogous R^2^ value was maintained. The results of the models were expressed as logarithms of *OR* with their respective *CI* and *P* values calculated according to the Knapp-Hartung method [23]. The existence of publication bias was investigated for each meta-analysis with sufficient number of studies using funnel plots, Egger’s test and the “trim and fill” method of Duval and Tweedie. Meta-analyses were performed using Comprehensive Meta-Analysis software V4 (Biostat, Englewood, New Jersey, USA), while descriptive characterizations were employed for variables that could not be submitted to meta-analyses.

### Analysis of the levels of evidence

The Grading of Recommendations, Assessment, Development and Evaluations (GRADE) tool was used to classify the associations submitted to meta-analyses by levels of evidence, also representing a novel approach in this review. The quality of evidence was interpreted based on GRADE methodology as follows: High evidence indicates a very high confidence that the true effect is close to the estimated effect; Moderate evidence suggests that the true effect is probably close to the estimate but could differ substantially; Low evidence reflects a significant likelihood that the true effect may differ considerably from the estimate; and Very low evidence indicates that the true effect is likely to differ substantially from the estimated effect [24–26].

Considering that all the articles analyzed referred to observational studies, the classification of level of evidence commenced at low [24] and was followed by an evaluation of several domains that could reduce or increase the level of evidence regarding the association observed. The domains that could lead to a reduction in the level of evidence included risk of bias, inconsistency, indirect evidence, imprecision, and publication bias, whereas those that could increase the classification comprised effect size, dose-response gradient, and the existence of non-controlled confounders that lead to underestimation of the association measure [25]. On this basis, the level of evidence for each of the associations was classified as high, moderate, low or very low [26]. Classifications were also established considering by grouping the variables socioeconomic/living conditions and previous occurrence of VL among household members, relatives or neighbors.

## Results

**Fig. 1:**
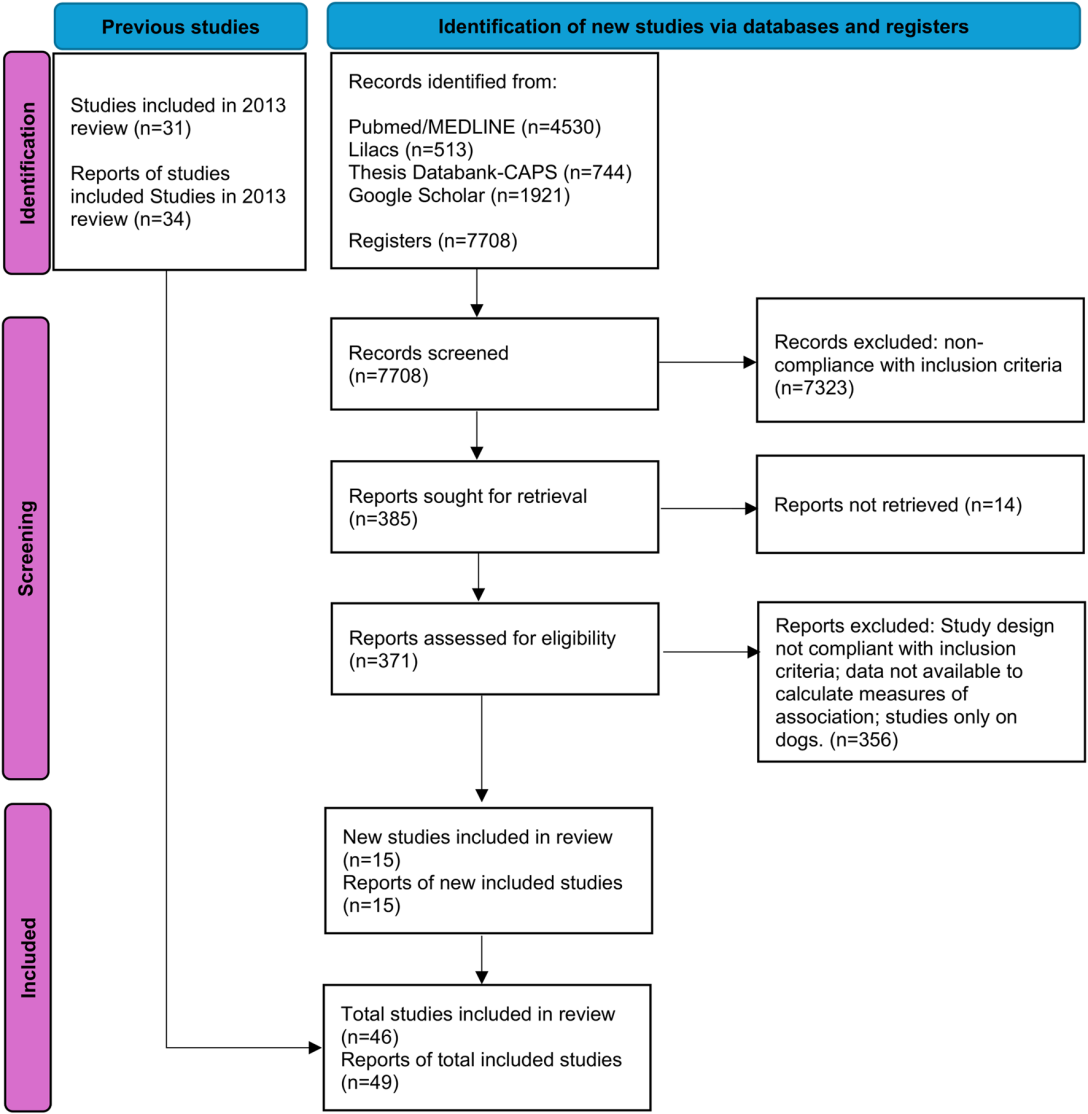
PRISMA flowchart showing the process of selection of articles included in the present review

The 2013 review [13] encompassed 31 individual level primary articles [27–57]. Fifteen new primary articles [14–16, 58–69] were included in the present review, affording a total of 46 publications. Considering that some articles include more than one study, a total of 49 studies were reviewed. Figure 1 shows the PRISMA flowchart summarizing the search and selection process. The study types were cross-sectional (*n* = 35), case-control (*n* = 10) and cohort (*n* = 4), the majority of which were conducted in Brazil (*n* = 38) with just a few in Venezuela (*n* = 3), Colombia (*n* = 2), Argentina, Mexico and Honduras (*n* = 1 for each country). The main diagnostic method employed was LST. Detailed information regarding each study is presented in the Additional file 3).

### Limitations and potential risks of bias of the selected studies

Two cohort studies were classified as low quality and two as moderate quality, whereas four case-control studies were classified as high quality, two as moderate quality, and four as low quality. Seven cross-sectional studies were classified as high quality, 15 as moderate quality and 13 as low quality (Additional file 2).

Comparison between the studies included in the 2013 review and the additional studies included in the present review revealed that quality had improved over the years. Among the cohort studies, the highest score (8 points) was attained by the only study of this type included in the new search. In the 2013 review, two of the seven (28.6%) case-control studies were classified as high quality, whereas in the present review two of the three (66.7%) recently included case-control studies were of high quality. The proportion of high-quality cross-sectional studies increased from 4.3% (1/23) in the 2013 review to 54.5% (6/11) among the recent studies. Additionally, the mean scores of case–control and cross-sectional studies increased, respectively, from 5.14 and 3.61 in the 2013 review to 8.3 and 6.4 among the newly included studies (Additional file 2). Although the quality of recent studies exhibited some improvement, especially regarding the control of confounders, shortcomings remain such as incomplete presentation of eligibility criteria, losses, refusals, lack of details about the procedures employed in building multiple models, and low statistical power of various associations (Additional file 3).

### Summary of the results of meta-analyses

Meta-analyses were conducted for 21 associations, seven of which had been performed in the 2013 review but were reconsidered with a larger number of studies, while four associations were newly identified with the inclusion of recent studies. Only the variable malnutrition evaluated in the 2013 review had no new studies added with the search update. Table 1 presents a comparative summary of the characteristics and main results of the meta-analyses conducted in the 2013 and present reviews.

**Table 1:** Comparison between the results of the 2013 review [13] and those of the present review for the variables of interest

Variables	Number of articles		Main findings		Level of evidence
	2013 review	Present review	2013 review	Present review	
Sex	18	27	Clinical studies and those involving LST diagnosis identified a significant positive association between men and VL, while studies involving serological tests revealed a significant inverse association	Studies involving LST diagnosis and serological tests established a positive association between men and VL, except that when serological tests were used the association was not statistically significant. Subgroup analysis was performed for different types of study and all of them identified a higher chance of VL in men. Strong and significant associations were described only in case-control studies	
Age Comparison: > 10-years old *vs.* < 10-years old	12	19	Clinical studies identified a strong positive and significant association between children and VL, while studies involving LST diagnosis established a significant inverse association	Studies involving LST diagnosis and serological tests identified a positive association between children and VL. Subgroup analysis was performed for different types of study but the higher chance of VL in children was identified only in case-control studies. In contrast, cross-sectional and cohort studies established an inverse association, which was statistically significant only in the first group	
Global age Comparison: < 10-years old *vs.* > 50-years old	No meta-analyses	8	Not applicable	Higher chance of VL in individuals > 50-years old but the association was not statistically significant	
Presence of dogs in the domicile	12	21	Weak but significant positive association when all studies were combined. Absence of significant heterogeneity among studies	Heterogeneities were identified. Subgroup analysis was performed for different types of study, which identified a positive association in cross-sectional and case control studies. However, the associations were strong and statistically significant only in the second group. Cohort studies identified inverse associations but without statistical significance	
Previous presence of seropositive dog in the domicile	No meta-analyses	4	Not applicable	Strong and positive associations but with occurrence of heterogeneities	
Presence of chickens/other fowl	8	15	Heterogeneities were not explained adequately by subgroups. No differences between the groups were detected when all study types were combined	Subgroup analysis was performed for different types of study, but positive and significant association was detected only in case-control studies. Other study types established inverse but not significant associations	
Presence of domestic/farm animals	No meta-analyses	Pigs (6), cats (5) and cattle/horses (4)	Not applicable	Weak and non-significant summary measures	
Previous contact with infected relatives or neighbors	Relatives (6) and neighbors (3)	Relatives (9) and neighbors (4)	Positive associations between VL and both groups, but with heterogeneities. Associations with relatives were stronger and statistically significant	Positive associations between VL and both groups, but associations with relatives were somewhat weaker, while associations with neighbors were slightly stronger	
Malnutrition	5	5 (Absence of new studies)	Weak and non-significant associations, with heterogeneities		
Backyard at the domicile or nearby	No meta-analyses	7	Not applicable	Positive weak associations with statistical significance, without heterogeneities	
Socioeconomic/living conditions	Treated water (3), sewage network (4), rubbish collection (3), floor covering (5), home finishing (8) and roof conditions (4)	Treated water (6), sewage network (6), rubbish collection (6), adequate floors (8), home finishing and roof conditions (14)	There were lower chances of VL when living conditions were good (valid for all variables). Occurrence of heterogeneities in most studies	There were lower chances of VL when living conditions were good. Subgroup analysis was introduced for home finishing and adequate floor covering, but subgroups did not explain adequately the heterogeneities	

*VL* visceral leishmaniasis, *LST Leishmania* skin test

Heterogeneities in the association between exposure and outcome variable were identified in most meta-analyses. The metrics (*P* values for Q test and *I*^2^ percentages) for all combinations and subgroups that showed explanatory capacities for the heterogeneities are presented in the Additional file 4. Age group and study period subgroups did not explain the heterogeneities of any of the associations.

### Results relating to the variables of interest

#### Sex

**Fig. 2:**
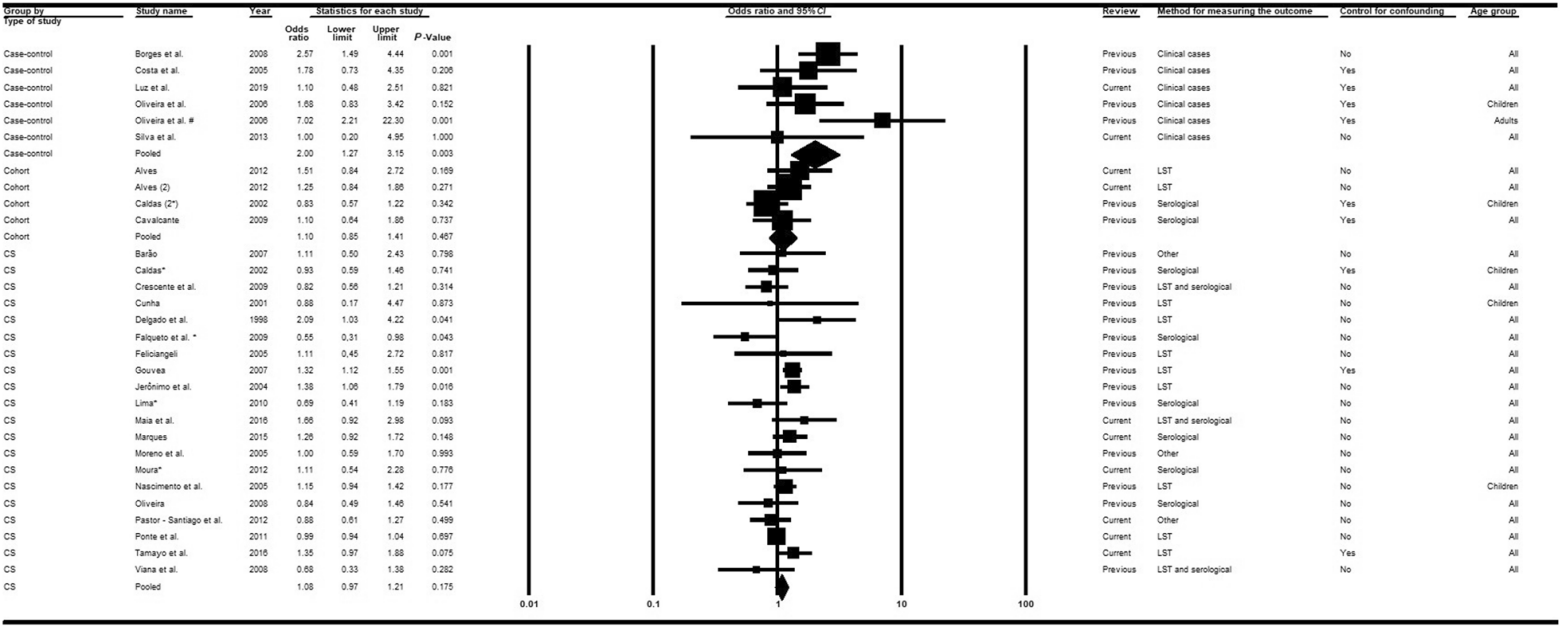
Forest plot for the variable sex in studies separated according to study type. *CS* cross-sectional, *LST Leishmania* skin test. Superscripts: (*) result of serological test in a study involving two diagnostic tests; (#) results in adults; (2) second result in a single article. The squares represent the weight of each study, whereas the diamonds represent the summary measurement of each subgroup. Category of reference: Females, Odds ratio = 1 [14, 15, 33, 36, 37, 39–43, 45–51, 53, 55–58, 60, 63, 65, 66, 68, 69]

**Fig. 3:**
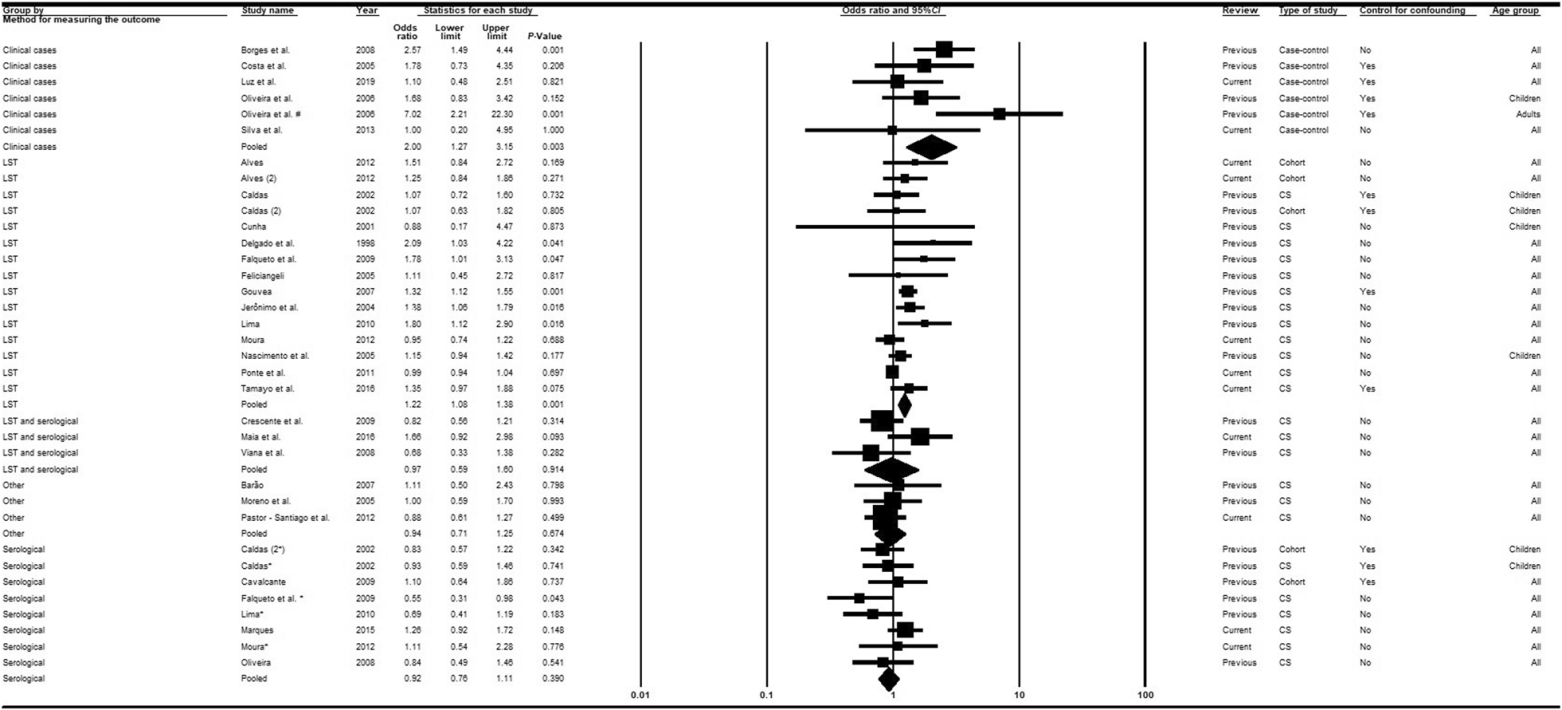
Forest plot for the variable sex in studies separated according to diagnostic method. *CS* cross-sectional, *LST Leishmania* skin test. Superscripts: (*) result of serological test in a study involving two diagnostic tests; (#) results in adults; (2) second result in a single article. The squares represent the weight of each study, whereas the diamonds represent the summary measurement of each subgroup. Category of reference: female, Odds ratio = 1 [14, 15, 33, 36–43, 45–51, 53, 55–58, 60, 63, 65, 66, 68, 69]

Twenty-seven articles [14, 15, 33, 36, 37, 39–43, 45, 46, 48–51, 53, 55–58, 60, 63, 65, 66, 68, 69] were analyzed, nine of which were recent publications [14, 15, 58, 60, 63, 65, 66, 68, 69]. Subgroup analyses considering the moderators study type (Fig. 2) and diagnostic method (Fig. 3) adequately explained the heterogeneities. When all epidemiological study types were combined, the chances of VL infection among men were higher than for women. In contrast, the combination of diagnostic methods indicated that the chances of VL among women were superior to men in studies involving serological tests (*OR* = 0.92; 95% *CI:* 0.76–1.11) or LST plus serological tests (*OR* = 0.97; 95% *CI:* 0.59–1.60). The associations were, however, generally weak, and not statistically significant except for case-control studies (*OR* = 2.00; 95% *CI:* 1.27–3.15) in which clinical manifestations associated with diverse diagnostic tests were used to confirm the outcome (Fig. 2). In the meta-regression analyses (Table 2), all groups exhibited a measure of association (negative log of the odds ratio values) that was significantly weaker than that obtained with the combination of case-control studies.

**Table 2:** Multiple meta-regression models of factors associated with human visceral leishmaniasis in the Americas based on moderators of study type, diagnostic methods, and control of confounders

Moderators/subgroups	Log *OR*	95% *CI*	*P* value^#^	R^2^
Sex				
Study types/cohort^§^	−0.60	−1.10, −0.11	**0.019**	0.32
Study types/cross-sectional	−0.63	−1.04, −1,21	**0.004**	
Diagnostic methods/LST^†^	−0.49	−0.9, −0.07	**0.023**	0.22
Diagnostic methods/LST and serological	−0.74	−1.28, −0.20	**0.009**	
Diagnostic methods/other tests	−0.75	−1.28, −0.22	**0.008**	
Diagnostic methods/serological	−0.79	−1.23, −0.35	**0.001**	
Control of confounders**/**yes^‡^	0.12	−0.16, 0.41	0.392	0.03
Age				
Study types/cohort	−3.81	−5.27, −2.34	** < 0.001**	0.65
Study types/cross-sectional	−3.84	−5.08, −2.61	** < 0.001**	
Diagnostic methods/LST	−4.12	−5.17, −3.07	** < 0.001**	0.88
Diagnostic methods/LST and serological	−3.72	−5.01, −2.43	** < 0.001**	
Diagnostic methods/other tests	−3.17	−4.28, −2.05	** < 0.001**	
Diagnostic methods/serological	−3.57	−4.66, −2.48	** < 0.001**	
Control of confounders**/**yes	−0.01	−1.38, 1.36	0.988	0.00
Presence of dogs in the domicile				
Study types/cohort	−0.90	−1.45, −0.35	**0.003**	0.64
Study types/cross-sectional	−0.62	−1.05, −0.19	**0.007**	
Diagnostic methods/LST	−0.64	−1.13, −0.15	**0.014**	0.17
Diagnostic methods/LST and serological	−0.54	−1.27, 0.19	0.135	
Diagnostic methods/other tests	−0.60	−1.16, −0.03	**0.039**	
Diagnostic methods/serological	−0.75	−1.33, −0.18	**0.013**	
Control of confounders**/**yes	−0.06	−0.38, 0.26	0.701	0.00
Presence of chickens/other fowl				
Study types/cohort	−0.66	−1.46, 0.14	0.101	0.22
Study types/cross-sectional	−0.69	−1.38, 1.01	**0.053**	
Diagnostic methods/LST	−0.72	−1.44, 0.01	**0.051**	0.00
Diagnostic methods/LST and serological	−0.59	−1.98, 0.8	0.373	
Diagnostic methods/other tests	−0.50	−1.29, 0.29	0.196	
Diagnostic methods/serological	−0,78	−1.51, −0.04	**0.041**	
Control of confounders**/**yes	−0.35	−0.73, 0.04	**0.073**	0.00
Study types/cohort**—**adjusted for control of confounders	−0.47	−1.31, 0.37	0.248	0.11
Study types/cross-sectional**—**adjusted for control of confounders	−0.54	−1.26, 0.17	0.126	
Control of confounders**/**yes—adjusted for study type	−0.24	−0.66, 0.18	0.245	

*LST* Leishmania skin test, *OR* odds ratio, *CI* confidence interval, *R*^*2*^ coefficient of determination

^#^Bold values are statistically significant

^**§**^Reference for study type: case-control studies

^†^Reference for diagnostic method: clinical cases

^‡^Reference for control of confounders: none

Analyses considering control of confounders indicated that the chances of VL among men were higher than for women in both subgroups (yes/no). No significant differences were identified between the association measures, although those in studies that employed control of confounders were slightly stronger (*OR* = 1.25; 95% *CI:* 1.00–1.59) compared to studies without such control (*OR* = 1.10; 95% *CI:* 0.97–1.25) and almost attained statistical significance (Additional file 5).

#### Age

**Fig. 4:**
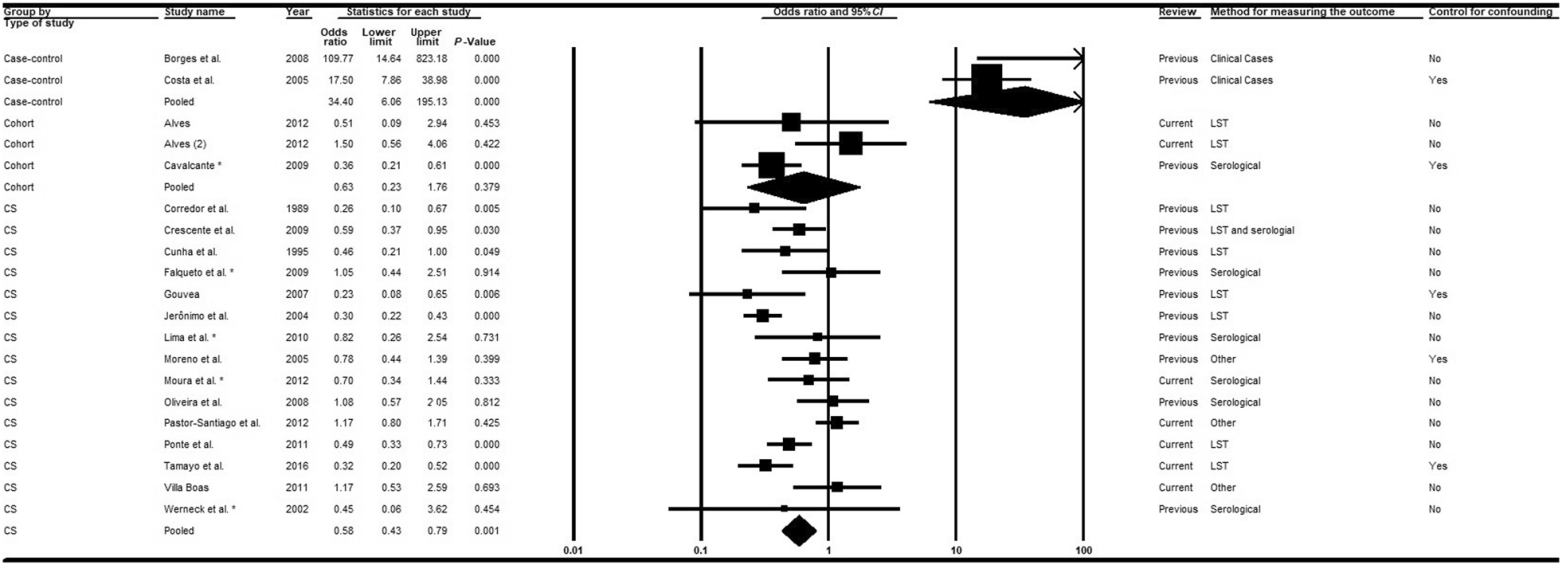
Forest plot for the variable age (< 10-years old to ≥ 10-years old) in studies separated according to study type. *CS* cross-sectional, *LST Leishmania* skin test. Superscripts: (*) result of serological test in a study involving two diagnostic tests; (#) results in adults; (2) second result in a single article. The squares represent the weight of each study, whereas the diamonds represent the summary measurement of each subgroup. Category of reference: ≥ 10 years of age, Odds ratio = 1 [14, 15, 28, 30, 38–40, 42, 48–50, 53, 55–60, 63]

**Fig. 5:**
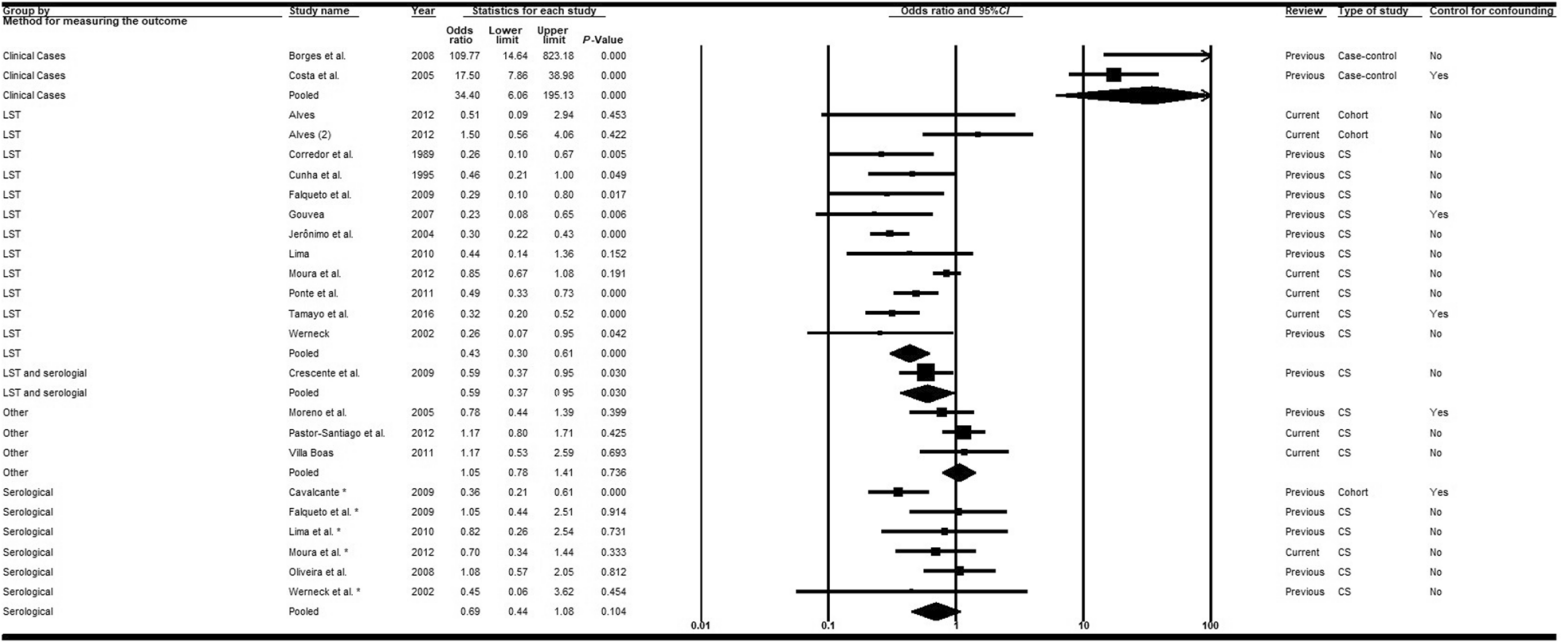
Forest plot for the variable age (< 10-years old to ≥ 10-years old) in studies separated according to diagnostic method. *CS* cross-sectional, *LST Leishmania* skin test. Superscripts: (*) result of serological test in a study involving two diagnostic tests; (#) results in adults; (2) second result in a single article. The squares represent the weight of each study, whereas the diamonds represent the summary measurement of each subgroup. Category of reference: ≥ 10 years of age, Odds ratio = 1 [14, 15, 28, 30, 38–40, 42, 48–50, 52, 55–60, 63]

Meta-analyses of the variable age were performed using < 10 years of age as the cut-off point as per the 2013 review. Since the results of studies involving children alone were not consistent [36, 37, 43, 54], our analysis focused on the remaining 19 articles [14, 15, 28, 30, 38–40, 42, 48–50, 53, 55–60, 63], six of which were recent publications [14, 15, 58–60, 63]. Analyses of studies according to study type and diagnostic method adequately explained the heterogeneities. The combination of case-control studies (clinical cases) showed the highest chances of VL among children under 10-year-old (*OR* = 34.40; 95% *CI:* 6.06–195.13) (Figs. 4 and 5), while chances of VL in this age group were lower in cross-sectional (*OR* = 0.58; 95% *CI:* 0.43–0.79) and cohort studies (*OR* = 0.63; 95% *CI:* 0.23–1.76) and with all other diagnostic criteria (Table 2). Analysis of the moderator diagnostic method (except for diagnosis by other tests) showed that there was an inverse association with lower chances of infection in children under 10-year-old (Figs. 4 and 5). Such associations were stronger and presented statistical significance in cross-sectional studies and with diagnosis by LST (*OR* = 0.43; 95% *CI:* 0.30–0.61). The measures of association were similar in studies both with (*OR* = 0.81; 95% *CI:* 0.22–2.96) and without control for confounders (*OR* = 0.78; 95% *CI:* 0.52–1.16) (Additional file 5).

**Fig. 6:**
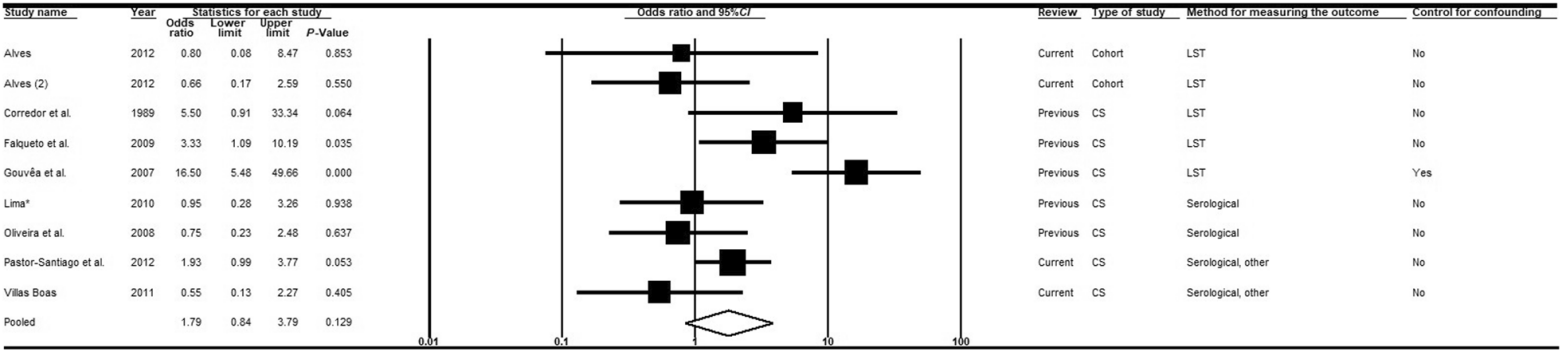
Forest plot for the variable age (< 10-years old to > 50-years old). *CS* cross-sectional, *LST Leishmania* skin test. Superscripts: (*) result of serological test in a study involving two diagnostic tests; (#) results in adults; (2) second result in a single article. The squares represent the weight of each study, whereas the diamonds represent the summary measurement of each subgroup. Category of reference: < 10 years of age, Odds ratio = 1 [14, 28, 48, 50, 56, 57, 59, 60]

An additional meta-analysis encompassing eight articles [14, 28, 48, 50, 56, 57, 59, 60] was performed to compare the chances of VL between > 50-year-old individuals and < 10-year-old children (Fig. 6). The chances of VL in the first group were higher compared with the second, although the association was not statistically significant (*OR* = 1.79; 95% *CI:* 0.84–3.79) and there were significant heterogeneities. Although it was not possible to carry out meta-analyses, some studies [33, 34, 41, 68] suggested that the proportion of infected individuals increased with age, particularly when diagnosis was by LST or serological tests.

#### Presence of dogs in the domicile

Eighteen articles [15, 27, 35, 37, 39, 42, 43, 48, 52, 53, 58–60, 63, 65, 67–69] were included in the meta-analyses of the variable presence of dogs in the domicile. Dissimilar from the 2013 review, the addition of nine recent articles [15, 58–60, 63, 65, 67–69] revealed heterogeneities in the association measures, and subgroup analyses and meta-regressions were performed. Subgroup analyses by study type showed significant heterogeneities within subgroups, but also the presence of heterogeneities between them (Additional file 4). Meta-regressions revealed that the R^2^ value for the moderator study type was approximately four-fold greater than for diagnostic method (Table 2) and, consequently, only the results of the first moderator are presented.

**Fig. 7:**
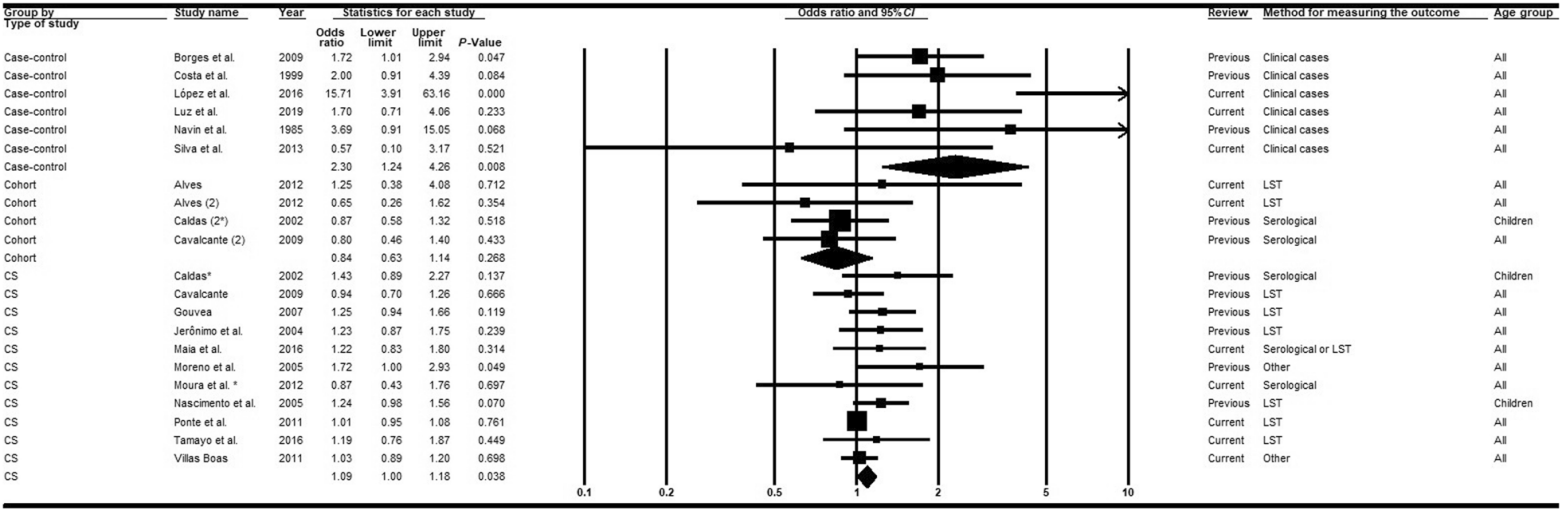
Forest plot for the variable presence of dogs in the domicile in studies separated according to study type. *CS* cross-sectional, *LST Leishmania* skin test. Superscripts: (*) result of serological test in a study involving two diagnostic tests; (#) results in adults; (2) second result in a single article. The squares represent the weight of each study, whereas the diamonds represent the summary measurement of each subgroup. Category of reference: absence of dogs, Odds ratio = 1 [15, 27, 35, 37, 39, 42, 43, 48, 52, 53, 58–60, 63, 65, 67–69]

Cross-sectional (*OR* = 1.09; 95% *CI:* 1.00–1.18) and case-control (*OR* = 2.30; 95% *CI:* 1.24–4.26) studies indicated a higher chance of VL in domiciles with dogs, although the associations were strong and statistically significant only in case-control studies. Inversely, but non-significant, associations were observed in cohort studies (*OR* = 0.84; 95% *CI:* 0.63–1.14) (Fig. 7). Studies involving diagnosis by LST, serological or other tests showed that the association measures were significantly weaker in comparison with clinical cases (in case-control studies) (Table 2).

In subgroup analysis by the control of confounders, associations between VL and the presence of dogs were demonstrated only in the combination of studies that did not control for such factors (*OR* = 1.22; 95% *CI:* 1.02–1.45) (Additional file 5). One of the cross-sectional studies [15] showed that the chances of VL increased with the number of dogs in the domicile (*OR* = 1.09; 95% *CI:* 1.87–1.38).

#### Presence of a seropositive dog in the domicile

**Fig. 8:**
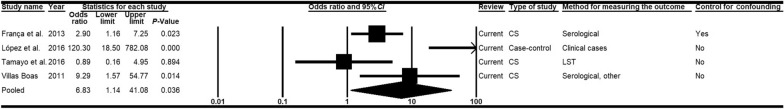
Forest plot for the variable previous presence of VL-positive dog. *CS* cross-sectional, *LST Leishmania* skin test; *VL* visceral leishmaniasis. Superscripts: (*) result of serological test in a study involving two diagnostic tests; (#) results in adults; (2) second result in a single article. The squares represent the weight of each study, whereas the diamonds represent the summary measurement of each subgroup. Category of reference: absence of VL-positive dog, Odds ratio = 1 [15, 59, 64, 67]

Four recent articles [15, 59, 64, 67] appraised the association between previous occurrence of canine VL in the domicile and the diagnosis of human VL. Combination of the obtained measures showed that the presence of a VL-positive dog was strongly and significantly associated with a higher chance of infection in humans (*OR* = 6.83; 95% *CI:* 1.13–41.08) (Fig. 8). However, significant heterogeneities were identified (Additional file 4) but it was not possible to perform subgroup analyses owing to the small number of studies.

#### Presence of chickens/other fowl at the domicile

**Fig. 9:**
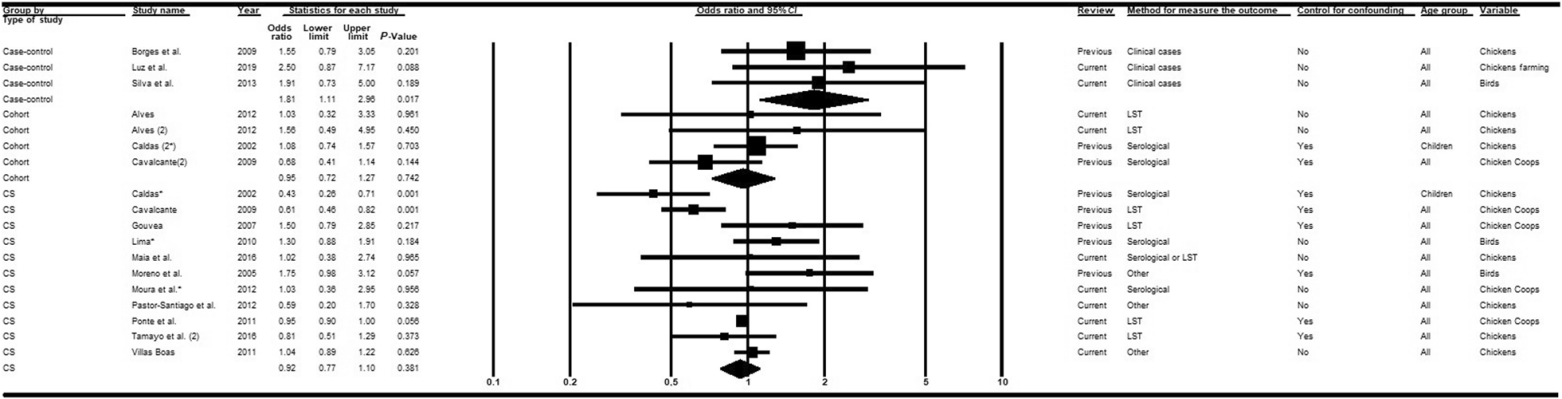
Forest plot for the variable presence of chickens/other fowl in studies separated according to study type. *CS* cross-sectional, *LST Leishmania* skin test. Superscripts: (*) result of serological test in a study involving two diagnostic tests; (#) results in adults; (2) second result in a single article. The squares represent the weight of each study, whereas the diamonds represent the summary measurement of each subgroup. Category of reference: absence of chickens/other fowl, Odds ratio = 1 [14, 15, 37, 42, 48, 52, 53, 57–60, 63, 65, 68, 69]

Fifteen articles [14, 15, 37, 42, 48, 52, 53, 57–60, 63, 65, 68, 69], nine of which [14, 15, 58–60, 63, 65, 68, 69] were recent publications included in the present review, investigated the association between VL and the presence of chickens/other fowl at the domicile. Unlike the results of the 2013 review, subgroup analyses of the moderator study type explained part of the heterogeneity in the association measures (Additional file 4). According to meta-regression models, only this moderator afforded explanatory capacity for the variance (R^2^ = 0.22; Table 2). Case-control studies showed a significantly higher chance of VL in domiciles with fowl (*OR* = 1.81; 95% *CI:* 1.11–2.96) (Fig. 9), whereas cohort (*OR* = 0.95; 95% *CI:* 0.72–1.27) and cross-sectional studies (*OR* = 0.92; 95% *CI:* 0.77–1.10) showed an inverse, but non-significant, association. Interestingly, there was a positive association in studies that did not perform control of confounders (*OR* = 1.11; 95% *CI:* 0.97–1.27) and an inverse association among those studies that did (*OR* = 0.86; 95% *CI:* 0.68–1.10) (Additional file 5).

Since the moderators study type and control of confounders showed a significant statistical association between VL and the presence of chickens/other fowl, multiple meta-regression models were constructed for this variable. The models showed that the strength of such association was weaker and no longer with statistical significance (Table 2).

#### Presence of domestic/farm/wild animals

The introduction of recent articles in the present review allowed the meta-analyses of associations between VL and other specific animals. Analyses of the presence of cats (*OR* = 1.18; 95% *CI:* 0.93–1.49) or a pig (*OR* = 0.95; 95% *CI:* 0.69–1.30) or horse/cattle (*OR* = 1.07; 95% *CI:* 0.91–1.26) enclosure demonstrated weak and non-significant associations without relevant heterogeneities (Additional file 6). Associations of VL with the presence of possums, bats, rats, armadillos, foxes and turtles were addressed in a single study [15, 68, 69], while associations with rabbits and monkeys were analyzed in two studies [15, 68].

#### Prior contact with infected household member, relatives or neighbors

**Fig. 10:**
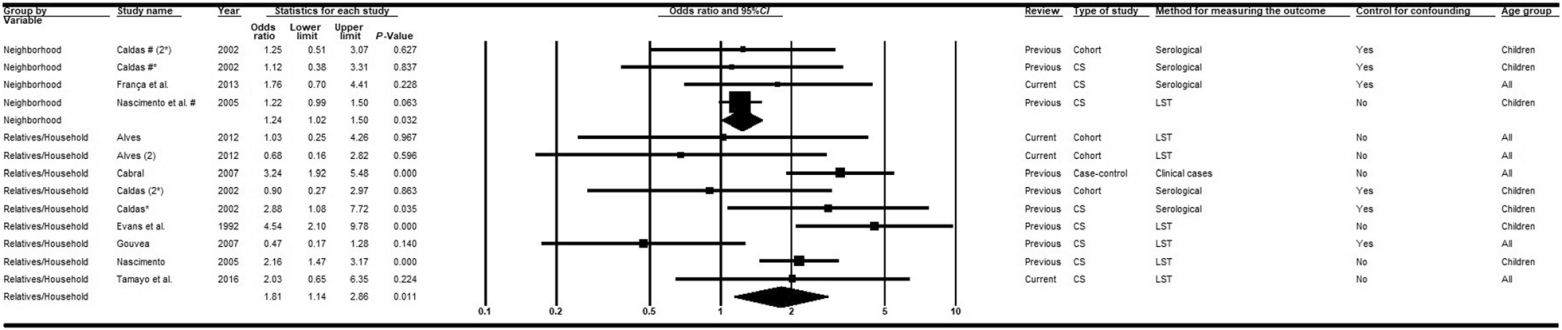
Forest plot for the variable occurrence of VL among household members, relatives or neighbors. *CS* cross-sectional, *LST Leishmania* skin test; *VL* visceral leishmaniasis. Superscripts: (*) result of serological test in a study involving two diagnostic tests; (#) results in adults; (2) second result in a single article. The squares represent the weight of each study, whereas the diamonds represent the summary measurement of each subgroup. Category of reference: absence of VL-positive household members, relatives or neighbors, Odds ratio = 1 [14, 29, 37, 43, 47, 48, 60, 64]

The relationship between VL and previous contact with infected relatives was examined in five studies [29, 37, 43, 47, 48], while contact with infected neighbors was investigated in three studies [14, 60, 64]. Meta-analyses showed significantly higher chances of VL in individuals who had prior contact with infected relatives (*OR* = 1.81; 95% *CI:* 1.14–2.86) or neighbors (*OR* = 1.24; 95% *CI:* 1.02–1.50) (Fig. 10), but heterogeneities were detected only in studies involving infected relatives (Additional file 4).

#### Socioeconomic/living conditions

Associations between VL and socioeconomic/living conditions were investigated in several studies using a diverse set of variables. Meta-analyses could be performed on nine different associations (Additional file 7), all of which indicated that individuals with better socioeconomic or living in domiciles with adequate infrastructure had lower chances of VL. Although heterogeneities were identified in associations involving most variables (Additional file 4), subgroup analyzes were performed only on the quality of home finishing or flooring, but no grouping adequately explained the heterogeneities. There was also a protective, but non-significant, association between VL and the presence of floor covering in studies with (*OR* = 0.60; 95% *CI:* 0.34–1.04) or without (*OR* = 1.79; 95% *CI:* 0.84–3.79) control of confounders, whereas a significant association between lower chances of VL and home finishing was identified only in studies that controlled confounders (*OR* = 0.68; *CI* 0.47–0.97) (Additional file 5).

Other socioeconomic variables have been appraised in the literature [14, 47, 58, 59, 65–67, 69] but only one or two studies were available for each variable. In general, the results followed the same pattern observed in the meta-analyses, i.e. adequate socioeconomic/living conditions minimized the chances of infection.

#### Accessible backyard at the domicile or nearby

**Fig. 11:**
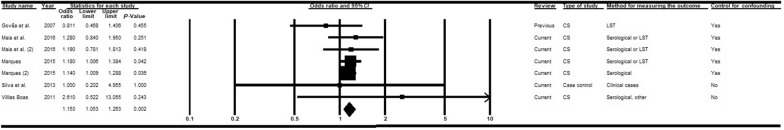
Forest plot for the variable presence of a backyard. *CS* cross-sectional, *LST Leishmania* skin test. Superscripts: (*) result of serological test in a study involving two diagnostic tests; (#) results in adults; (2) second result in a single article. The squares represent the weight of each study, whereas the diamonds represent the summary measurement of each subgroup. Category of reference: absence of backyard, Odds ratio = 1 [48, 59, 65, 66, 68]

Five articles assessed the association between VL and the presence of a backyard at the domicile or in the neighborhood [48, 59, 65, 66, 68] (Fig. 11). Meta-analyses of the combined studies indicated a positive weak and significant association (*OR* = 1.15; 95% *CI:* 1.05–1.26) and there was no heterogeneity between the measures of association. The results of one case-control study [69] revealed associations between VL and the presence of a vegetable garden (*OR* = 5.3; 95% *CI:* 1.3–21.2), a yard with fruit trees (*OR* = 5.2; 95% *CI:* 1.5–18.6), a yard with non-fructiferous trees (*OR* = 7.2; 95% *CI:* 2.4–21.3), a yard with fruits on the ground (*OR* = 10.7; 95% *CI:* 2.0–52.1), or a yard with leaves on the ground (*OR* = 3.5; 95% *CI:* 1.3–9.0). Another study [28] recorded an association between the increased likelihood of infection and backyards that combined characteristics such as infrequent cleaning, presence of trees, waste, ants, and other insects.

Associations between VL and the presence of vegetation, such as the presence of a tree on the pavement in front of the house, a tree within a 10 m radius of the residence, a forest near the residence, a sidewalk/patio with shade or fruit trees, plants inside the house, plants outside the house, or plants and garden close to the house were each evaluated in single studies [37, 44, 57, 60, 62, 65, 67, 69].

#### Other variables

Since no new articles evaluating the potential role of malnutrition on the likelihood of asymptomatic infection by *L. infantum* were available, the previous conclusion that there is no association between VL infection and malnutrition remains unchanged [13].

For many variables, it was not possible to combine the data into summary measures because of the differences among the aspects analyzed and insufficient numbers of studies. Such variables included knowledge about VL, daily habits (working or studying away from home), sitting on the sidewalk in front of the house during the evening, staying outside the house after 6:00 pm, staying inside the house between 6:00 and 10:00 pm on hot days, the presence of sandflies, location of the bath/shower, location of the television, presence of shaded areas, presence of humid areas, recreational activities, having been born in a town with autochthonous VL cases, the presence of fly breeding sites, sleeping habits (sleeping at night with the window open, or with a fan or air conditioning turned on), the proximity of the house to buildings, parks or squares, the location of animal waste, the presence of a dog confined to a kennel overnight or always free in the yard, time spent in the company of a dog, the frequency of yard cleaning, previous blood transfusion, and overweight/obesity [32, 38, 58, 60, 61, 63, 64, 66–68].

### Publication bias

**Fig. 12:**
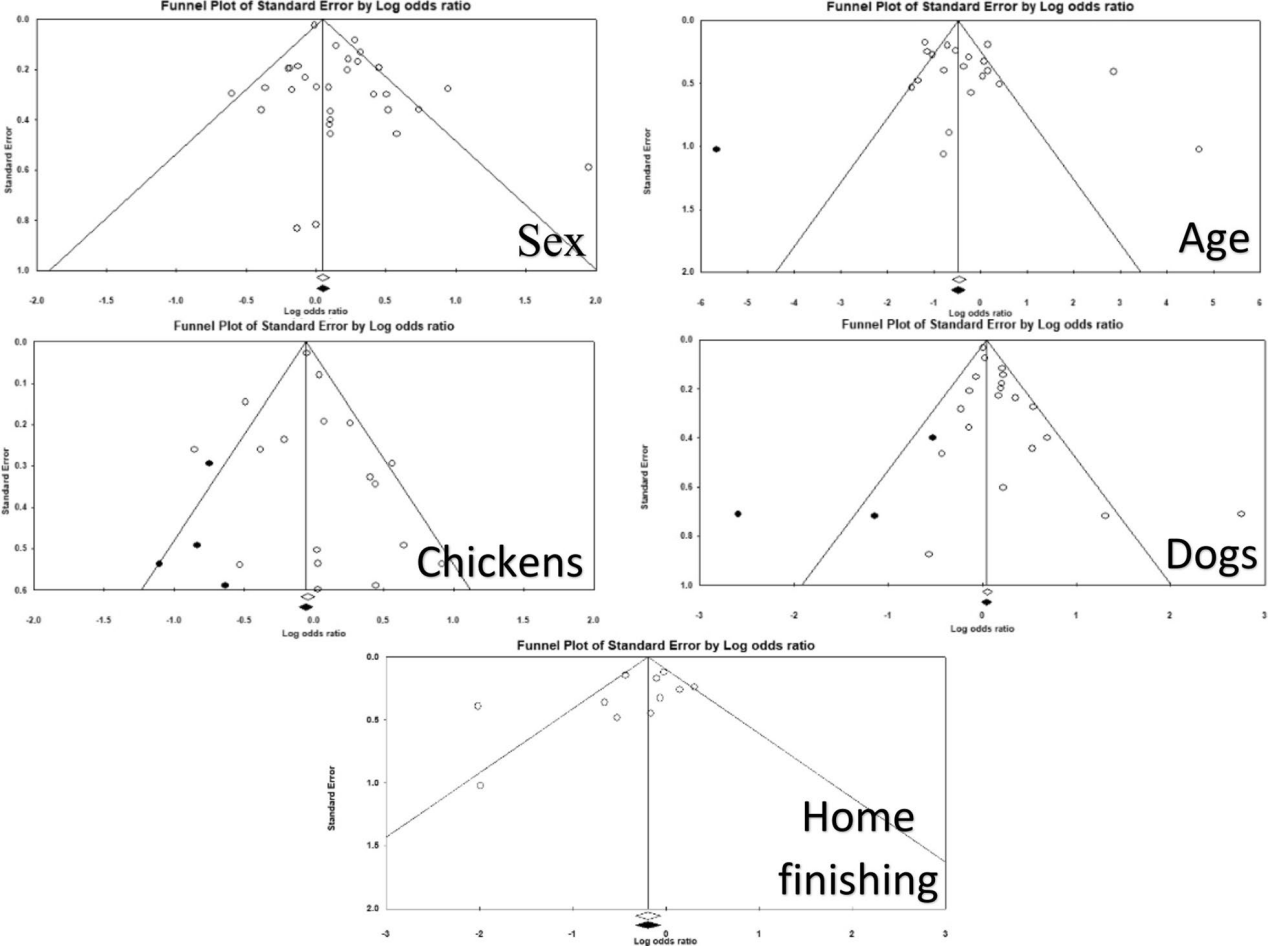
Funnel plots for the variables sex; age; presence of chickens/other fowl at the domicile; presence of dogs in the domicile, and home finishing. Closed dots correspond to imputed studies using the “trim and fill” method of Duval and Tweedie

The presence of publication bias was assessed for five VL associations (Fig. 12). For the variables sex and home finishing, the Egger test showed that funnel plot asymmetry was not significant (*P* < 0.05) suggesting the absence of publication bias, hence no studies were imputed in the funnel plot using the “trim and fill” method. For the variables age (< or ≥ 10 years old) and presence of chickens/other fowl, the Egger test showed that funnel plot asymmetry was not significant (*P* < 0.05), although studies were imputed to obtain symmetry. Despite this procedure, the summary measure remained unchanged. For the variable presence of dogs in the domicile, the Egger test demonstrated the existence of significant publication bias (*P* = 0.01). After imputation of three studies by the “trim and fill” method the measure of association was slightly reduced, and it was no longer significant.

### Levels of evidence

The final classification of the associations described in this review by levels of evidence is shown in Table 1, with more detailed information presented in the Additional file 8. The level of evidence for most of the associations was classified as low or very low. However, the estimates for the variables sex and age (< 10 years old vs > 10 years old) at the initial level of classification not only received no downgrades but was upgraded by one factor. Thus, these variables were classified as moderate level of evidence.

## Discussion

This systematic review expands our understanding of the factors associated with human VL in the Americas through the inclusion of articles published since June 2011, the cut-off point used in a previous review [13]. Moreover, we have employed additional methods of analysis to strengthen the conclusions about the associations and sources of heterogeneity, and to assess the progression in quality of recent publications. Updating the factors associated with human VL has allowed us to propose new directions for future research.

### Associations

The results obtained from all types of epidemiological studies have shown that VL is more common among men than women. Since the association was significant and strong only in case-control studies, it can be concluded with a moderate level of evidence that men have a higher chance of developing symptomatic forms of the disease. This level of evidence was supported by the magnitude of the effect and its clinical relevance. Moreover, the conclusion is reinforced by the high proportion of infected males (63% of cases) notified to the Brazilian health services [70]. On the other hand, regarding the chance of infection, it is not possible to draw definitive conclusions. According to some authors, there may be a greater probability of infection among males, which could be attributed to a combination of behavioral and social factors [71–73], whereas clinical manifestations of the disease may be related to genetic, hormonal, and immunological factors [74–77]. In the previous [13] and in the present review, however, the association between VL and gender was in the opposite direction in studies using serological tests, with males showing a lower chance of VL. The variety of diagnostic methods used for assessing VL may capture a range of conditions related to the gender associated risk of infection and disease, from immunological to social and cultural features [76–79].

Age also influences the susceptibility to VL in that the disease is more common among young children and the elderly [70]. The combined results of case-control studies showed a strong and significantly higher chance of VL among children aged < 10 years, the level of evidence for which was classified as moderate, following the same criteria applied to the sex variable. Such vulnerability can be attributed to an immature immune system that is often aggravated by malnutrition [4, 80, 81]. On the other hand, the results from the combination of cohort and cross-sectional studies indicated that, despite the manifestation of symptoms, the likelihood of infection may be lower among children. Furthermore, a new meta-analysis included in the present review, which involved eight studies comparing the occurrence of VL in children and older adults showed that individuals over 50 years of age had a higher chance of being infected than children under 10 years old. However, considering that only three cohort studies were included in our meta-analysis and laboratory tests remain positive long after the onset of infection, it cannot be concluded with certainty that children present a lower probability of acquiring the infection.

In the 2013 review, a single meta-analysis indicated that the probability of VL was slightly higher in domiciles in which dogs were present compared with those that had no dogs. In the present review, the association between VL and the presence of dogs was strong and significant only in case-control studies, whilst in cohort studies the direction of the association was reversed. The complexity of the disease transmission cycle, including the influence of alternative reservoirs, environmental and climatic conditions across different regions, socioeconomic factors, and the level of care provided by dog owners, should be taken into account in this interpretation [82]. Moreover, the inclusion of new studies from different areas is expected to modify and may further alter the existing evidence. In view of the very low level of evidence available and noting that the results obtained in case-control studies could be explained by recall bias [83], it cannot be concluded that the presence of dogs increases the chance of infections by *L. infantum*. Future studies should aim to overcome methodological limitations through prospective designs, improved standardization of data collection, and rigorous control of confounding factors.

There was a positive association between human VL and the previous presence of a seropositive dog in the domicile despite the small number of studies included in the meta-analysis which led to the evidence being classified as very low for the effect measure. On the other hand, since dogs are the main reservoirs of VL in urban environments [4], it is plausible to conclude that the presence of infected dogs increases the risk of human infection [17, 84].

Although with a level of evidence classified as low due to the small number of studies and the presence of heterogeneities, the results of the analyses reported herein reinforce the conclusion identified in the earlier review [13] that previous cases of VL among household members, relatives or neighbors increase the chances of the disease in healthy individuals. This association is highly plausible and can be explained by factors such as geographic proximity, sharing environments and similar habits [63].

The present review supports previous declarations of a lack of evidence of a relationship between VL and the presence of chickens/other fowl at the domicile [13, 85]. Such an association was indicated only by case-control studies, a finding that can likely be explained by recall bias. Furthermore, other types of epidemiological studies and those that controlled confounders, found the association to be in the opposite direction. Nevertheless, this issue should be studied further, especially through cohort studies, and expanded to investigate the role of chickens/other fowl in the acquisition of infection not only by members of the household but by those of neighboring domiciles [86].

Associations between VL and other domestic/farm/wild animals in general have not been widely investigated, and meta-analyses could only be performed with studies focusing on the presence of cats, pigs and horses/cattle. The results of this evaluation indicated that the association measures were weak, not statistically significant and heterogeneous. Thus, whilst it is acknowledged that the presence of animals at the domicile generates considerable amounts of organic waste that favors the breeding of sandflies [87], there is insufficient evidence to state that the presence of any domestic animals increases the probability of VL.

The association between VL and access to a backyard at the domicile was better investigated in the present review, and the results revealed that there was a slightly higher chance of infection in households with backyards compared with those without, although the level of evidence was low due to the absence of factors that could elevate the strength of evidence for the association analyzed in observational studies. Backyards provide a favorable environment for the proliferation of sandflies owing to the presence of vegetation, shade, humidity and the accumulation of organic waste [88, 89].

The present review included a large number of studies that analyzed the relationship between different aspects associated with socioeconomic/living conditions and human VL at the individual level. Considering the pattern of results for all variables, it was possible to conclude that VL is associated with poor standards of living, a relationship that has been amply demonstrated in ecological studies [85, 90, 91] showing that precarious and inadequate housing favor the advent and maintenance of the disease.

### Directions for preventive and control actions

It is important to highlight the need to expand the use of existing knowledge about the factors associated with VL in directing prevention and control actions [4]. In this context, targeted measures directed at dogs cannot be dismissed. This is reinforced by the results obtained from the analysis of the association between the presence of positive animals and the risk of infection. Euthanasia of seropositive dogs is still employed in Brazil to control the spread of VL [4] even though the practice has proven to be less effective [92, 93] due mainly to the replacement of euthanized dogs by other infected animals [94, 95]. One of the recommended alternatives to euthanasia involves the use insecticide-impregnated collars [96, 97], which can be effective if appropriately targeted in defined geographical areas [84] or towards groups of individuals considered to be priorities. The obtained results indicate that special attention should be given by health services not only to infected dogs that have not been euthanized due to the refusal of the guardians, but also to those animals that continue to coexist with them or even to those that have replaced the euthanized dogs [5, 98–100]. Insecticide collars are one of the measures that could be prioritized for these animals.

Findings regarding previous cases of VL among household members, relatives or neighbors also highlights the importance of specific interventions at transmission foci [90] as well as the need for epidemiological surveillance programs capable of monitoring outbreaks of *L. infantum* infection and directing appropriate and timely actions in areas close to where cases occur [4].

Considering that age certainly influences the symptoms of VL and the manifestations are more severe in children and in the elderly [10], it is important to prioritize these groups with respect to preventive care and reduction of lethality [81, 84]. Educational campaigns could also focus on men, who exhibit higher incidences of symptomatic VL. With regard to backyards, while the association with VL should be further investigated, it is worthwhile recommending the regular cleaning of backyards and pruning of the trees in VL transmission areas [88, 89]. Additionally, vector and reservoir control measures could be prioritized in households with poor economic conditions or for individuals living with seropositive dogs or with prior contact with infected animals, household members, relatives, or neighbors.

Finally, the findings related to socioeconomic factors reinforce the importance of reducing economic inequalities and the need to strengthen the integration of actions to control VL with those aimed at other neglected tropical diseases [86, 87].

The impact of these and other strategies based on the associations studied should be assessed in intervention studies regarding the occurrence of human and canine VL [101, 102].

### Quality of publications and directions for future studies

One of the positive aspects identified in the present review was the number of recent articles presenting improved quality due mainly to the expansion of procedures for controlling confounding effects. Failure to account for these confounding factors can lead to inadequate estimates and misinterpretation of associations. In this context, and considering the complexity and diversity of risk and protective factors for VL, future research should collect information about broad sets of explanatory variables and implement appropriate procedures to control confounders. Additionally, the relationships between the different variables should be analyzed using methodologies such as causal diagrams. These tools are essential for identifying causal relationships by visually representing the pathways through which variables interact and influence one another and are particularly valuable in guiding the selection of variables to be adjusted in statistical models. Furthermore, structural equation models and artificial intelligence techniques, like causal diagrams, which have also not yet been utilized in this subject area, should be adopted to enhance analytical approaches and provide deeper insights into the determinants of VL [103–107].

It is important to note that, despite the positive evolution, the methodological quality of the studies was still limited and the level of evidence for most associations was considered to be low or very low. Thus, our review highlights the uncertainty of the majority of the effect estimates obtained and the need for further improvements in the methodological quality of the studies. However, it is important to emphasize that the tenets of the GRADE approach prompt an initial low-level classification of evidence from observational studies because of the impossibility of randomization and the inherent presence of residual confounding [24]. Conversely, except for the association between VL and the presence of chickens/other fowl, there were no relevant differences regarding the association estimates between studies with and without control of confounders. Hence, the interpretation of level of evidence must take into consideration the subjectivity and criteria of the GRADE approach together with the intrinsic limitations of observational studies. In brief, evaluation of the levels of evidence must be contextualized.

In addition to the recommendations regarding strategies for controlling confounding factors and establishing causal relationships, it is essential that future research on VL be conducted with greater statistical power, in diverse Latin American countries and preferably through cohort studies. Regarding diagnostic methods, research should prioritize the use of molecular techniques, such as polymerase chain reaction (PCR), in combination with serological tests, given the necessity of employing multiple methodologies to confirm the diagnosis [108, 109]. Molecular biology techniques have demonstrated superior accuracy in diagnosing VL. Nonetheless, their widespread application is hindered by the requirement for advanced technological infrastructure and extended processing times [109]. Hence, it is imperative to allocate funding to research in this field, enabling broader implementation of molecular diagnostic methods and advancing their accessibility. Additionally, the use of rapid diagnostic tests, which offer significant operational advantages, should also be encouraged [108, 109]. Since diagnostic methods can affect the associations between risk factors and outcomes (as observed with gender associations), future studies should also consider this aspect in study design and result interpretation. Moreover, a broader evidence base is needed to improve the understanding of these influences in future systematic reviews.

### Limitations

The major limitations of the present review are the combination of adjusted and unadjusted association measures and those that had been adjusted for different variables. The ideal strategy would be to combine studies that were as similar as possible in terms of the control of confounders and other aspects, but the low number of available publications and the complexity of the set of variables studied render such an approach unfeasible. Even with the increased numbers of studies included in the present review, very few results were available for several important variables and in-depth evaluation could not be performed. Among these potentially relevant issues, the association of VL with the presence of phlebotomies in the domicile, the sleeping habits of individuals, and knowledge about the disease are aspects that demand further investigation.

Other limitations mentioned in the 2013 review [13] may also have impacted on the results such as the impossibility of performing subgroup and publication bias analyses for some associations and of obtaining summary measures with heterogeneity. On the other hand, the possibility of publication bias was considered to be minor for most indicated associations with the exception of that between VL and the presence of dogs in the domiciles.

## Conclusions

The periodical update of evidence emerging from new studies is important for augmenting our understanding of the dynamics of a disease [110], a viewpoint that is validated by the contribution of our present review in which several relevant aspects of VL are clarified and various new questions raised. Although men and children exhibit a higher incidence of symptomatic visceral leishmaniasis, the probability of infection appears consistent across these groups. Moreover, there is a lack of sufficient evidence to substantiate the claim that having dogs or fowl in the household elevates the risk of VL. However, socioeconomic and living conditions, as well as prior occurrences of human and canine VL, are significant contributors. The review findings also underscore the importance of enhanced study designs. This includes adopting cohort studies, ensuring sample sizes with sufficient statistical power, utilizing appropriate diagnostic and analytical methods, and incorporating innovative techniques, such as causal diagrams, to elucidate complex relationships among variables. Updating the factors associated with human VL is essential for designing more effective prevention and control strategies and reducing the impact of the disease on vulnerable populations.

## Supplementary Information

Additional file 1. Terms used to search for publications

Additional file 2. Analysis of the methodological quality of the studies included using the Joanna Briggs Institute appraisal tools

Additional file 3. Characteristics and limitations of studies included in the systematic review

Additional file 4. Description of Q values, *P* values and *I²*.

Additional file 5. Forest plots for variables with subgroup analyses by controlling for confounding. Fig. S1 Forest plot for the sex variable: studies divided into subgroups by confounding control. Abbreviations: *CS* cross-sectional; *LST* *Leishmania* skin test. Superscripts:result of serological test in a study involving two diagnostic tests;results in adults;second result in a single article. The squares represent the weight of each study, whereas the diamonds represent the summary measurement of each subgroup. Reference: Female, Odds ratio = 1. [14, 15, 33, 36, 37, 39–41, 42, 43, 45, 46, 48 – 51, 53, 55–58, 60, 63, 65, 66, 68, 69]. Fig. S2 Forest plot for the age variable: studies divided into subgroups by confounding control. Abbreviations: *CS* cross-sectional; *LST* *Leishmania* skin test. Superscripts:result of serological test in a study involving two diagnostic tests;results in adults;second result in a single article. The squares represent the weight of each study, whereas the diamonds represent the summary measurement of each subgroup. Reference: Being over 10 years old, Odds Ratio = 1. [14, 15, 28, 30, 38–40, 42, 48–50, 53, 55–60, 63]. Fig. S3 Forest plot for the variable presence of dog in the domicile: studies divided into subgroups by confounding control. Abbreviations: *CS* cross-sectional; *LST* *Leishmania* skin test. Superscripts:result of serological test in a study involving two diagnostic tests;results in adults;second result in a single article. The squares represent the weight of each study, whereas the diamonds represent the summary measurement of each subgroup. Reference: Not having dogs, Odds Ratio = 1. [15, 27, 35, 37, 39, 42, 43, 48, 52, 53, 58–60, 63, 65, 67–68]. Fig. S4 Forest plot for the variable presence of chickens/other fowl at the domicile: studies divided into subgroups by confounding control. Abbreviations: *CS* cross-sectional;* LST Leishmania* skin test. Superscripts:result of serological test in a study involving two diagnostic tests;results in adults;second result in a single article. The squares represent the weight of each study, whereas the diamonds represent the summary measurement of each subgroup. Reference: Do not have chickens and poultry, Odds Ratio = 1. [14, 15, 37, 42, 48, 52, 53, 57 – 60, 63, 65, 68, 69]. Fig. S5 Forest plot for the floor variable: studies divided into subgroups by confounding control. Abbreviations: *CS* cross-sectional; *LST* *Leishmania* skin test. Superscripts:result of serological test in a study involving two diagnostic tests;results in adults;second result in a single article. The squares represent the weight of each study, whereas the diamonds represent the summary measurement of each subgroup. Reference: Inadequate, Odds Ratio = 1. [14, 40, 43, 48, 53, 57, 59, 60]. Fig. S6 Forest plot for the variable house finishing: studies divided into subgroups by confounding control. Abbreviations: *CS* cross-sectional; *LST* *Leishmania* skin test. Superscripts:result of serological test in a study involving two diagnostic tests;results in adults;second result in a single article. The squares represent the weight of each study, whereas the diamonds represent the summary measurement of each subgroup. Reference: Inadequate, Odds Ratio = 1. [14, 37, 40, 42, 43, 48, 53, 63].

Additional file 6. Forest plot for the variable presence of other animals at the domicile:cattle/horses;cats;pigs. Abbreviations: *CS*,cross-sectional; *LST* *Leishmania* skin test. Superscripts:result of serological test in a study involving two diagnostic tests;results in adults;second result in a single article. The squares represent the weight of each study, whereas the diamonds represent the summary measurement of each subgroup. Category of reference: absence of pigs, cats and cattle/horses, Odds ratio = 1. References: [14, 15, 58, 59, 63, 65, 68, 69].

Additional file 7. Forest plots of socioeconomic variables associated with human visceral leishmaniasis in the Americas. Fig. S1 Forest plot for the water supply variable. Abbreviations: *CS* cross-sectional; *LST* *Leishmania* skin test. Superscripts:result of serological test in a study involving two diagnostic tests;results in adults;second result in a single article. The squares represent the weight of each study, whereas the diamonds represent the summary measurement of each subgroup. Reference: Non-piped water, Odds Ratio = 1. [40, 43, 48, 60, 63, 65]. Fig. S2 Forest plot for the sewage system variable. Abbreviations: *CS* cross-sectional; *LST* *Leishmania* skin test. Superscripts:result of serological test in a study involving two diagnostic tests;results in adults;second result in a single article. The squares represent the weight of each study, whereas the diamonds represent the summary measurement of each subgroup. Reference: Absence of sewage system, Odds Ratio = 1. [40, 48, 53, 63, 69]. Fig. S3 Forest plot for the garbage collection variable. Abbreviations: *CS* cross-sectional; *LST* *Leishmania* skin test. Superscripts:result of serological test in a study involving two diagnostic tests;results in adults;second result in a single article. The squares represent the weight of each study, whereas the diamonds represent the summary measurement of each subgroup. Reference: Public garbage collection, Odds Ratio = 1. [40, 42, 53, 63, 65, 67]. Fig. S4 Forest plot for the floor variable. Abbreviations: *CS* cross-sectional; *LST* *Leishmania* skin test. Superscripts:result of serological test in a study involving two diagnostic tests;results in adults;second result in a single article. The squares represent the weight of each study, whereas the diamonds represent the summary measurement of each subgroup. Reference: Inadequate, Odds Ratio = 1. [14, 40, 43, 48, 53, 57, 59, 60]. Fig. S5 Forest plot for the house finishing variable. Abbreviations: *CS* cross-sectional; *LST* *Leishmania* skin test. Superscripts:result of serological test in a study involving two diagnostic tests;results in adults;second result in a single article. The squares represent the weight of each study, whereas the diamonds represent the summary measurement of each subgroup. Reference: Inadequate, Odds Ratio = 1. [14, 37, 40, 42, 43, 48, 53, 63]. Fig. S6 Forest plot for the income variable. Abbreviations: *CS* cross-sectional; *LST* *Leishmania* skin test. Superscripts:result of serological test in a study involving two diagnostic tests;results in adults;second result in a single article. The squares represent the weight of each study, whereas the diamonds represent the summary measurement of each subgroup. Reference: More than 01 minimum wage, Odds Ratio = 1. [45, 59, 69]. Fig. S7 Forest plot for the variable education. Abbreviations: *CS* cross-sectional; *LST* *Leishmania* skin test. Superscripts:result of serological test in a study involving two diagnostic tests;results in adults;second result in a single article. The squares represent the weight of each study, whereas the diamonds represent the summary measurement of each subgroup. Reference: Some education, Odds Ratio = 1. [45, 49, 59, 66]. Fig. S8 Forest plot for the variable education. Abbreviations: *CS* cross-sectional; *LST* *Leishmania* skin test. Superscripts:result of serological test in a study involving two diagnostic tests;results in adults;second result in a single article. The squares represent the weight of each study, whereas the diamonds represent the summary measurement of each subgroup. Reference: Non-elementary, Odds Ratio = 1. [40, 48, 60]. Fig. S9 Forest plot for the variable number of residents per household. Abbreviations: *CS* cross-sectional; *LST* *Leishmania* skin test. Superscripts:result of serological test in a study involving two diagnostic tests;results in adults;second result in a single article. The squares represent the weight of each study, whereas the diamonds represent the summary measurement of each subgroup. Reference: < 4 people, Odds Ratio = 1. [14, 40, 42, 53, 59].

Additional file 8. Results of analyses and interpretation of the level of evidence using the GRADE approach. Table S1. Interpretation of the quality of evidence according to the GRADE methodology. Table S2. Results of the GRADE approach by domains

## Data Availability

All data generated or analyzed during this study are included in the published article and Supplementary Material. Any extra information is available from the corresponding author on reasonable request.

## Abbreviations

*CI*: Confidence interval
CS: Cross-sectional
GRADE: Grading of recommendations, assessment, development and evaluations
LST: *Leishmania* skin test
MOOSE: Meta-analysis of observational studies in epidemiology
*OR*: Odds ratio
PRISMA: Preferred reporting items for systematic reviews and meta-analyses
VL: Visceral leishmaniasis
