# Supplementary material for: An updated systematic review with meta-analysis and meta-regression of the factors associated with human visceral leishmaniasis in the Americas
Source: Infect Dis Poverty. 2025 Jan 30;14:4. doi: 10.1186/s40249-025-01274-z (PMC11781006; doi:10.1186/s40249-025-01274-z)
Supplement: Supplementary file 1 — Additional file 1. Terms used to search for publications [file 40249_2025_1274_MOESM1_ESM.docx]

**Additional file 1 - Terms used to search for publications (keywords and MeSH)**

**PubMed:** (Leishmaniasis, Visceral OR Leishmania infantum) AND (risk factors OR associated factors OR epidemiological studies OR immunology OR epidemiology) **LILACS:** (visceral leishmaniasis OR leishmaniose visceral AND risk factors OR immunology); **CAPES Databank:** leishmaniose visceral AND epidemiol*; e **Google Scholar:** allintitle: Leishmaniose visceral
