# Supplementary material for: An updated systematic review with meta-analysis and meta-regression of the factors associated with human visceral leishmaniasis in the Americas
Source: Infect Dis Poverty. 2025 Jan 30;14:4. doi: 10.1186/s40249-025-01274-z (PMC11781006; doi:10.1186/s40249-025-01274-z)
Supplement: Supplementary file 2 — Additional file 2. Analysis of the methodological quality of the studies included using the Joanna Briggs Institute appraisal tools [file 40249_2025_1274_MOESM2_ESM.docx]

**Additional file 2: Analysis of the methodological quality of the studies included using the Joanna Briggs Institute appraisal tools**

**Cohort Studies**

| **First Author/ Year** | **Title** | **Review** | **Q1** | **Q2** | **Q3** | **Q4** | **Q5** | **Q6** | **Q7** | **Q8** | **Q9** | **Q10** | **Q11** | **Total** | **Quality** |
| --- | --- | --- | --- | --- | --- | --- | --- | --- | --- | --- | --- | --- | --- | --- | --- |
| Alves et al. / 2016 | Fatores de risco para incidência de infecção por *Leishmania infantum* na cidade de Teresina, Piauí | Current | **Y** | **Y** | **Y** | **Y** | **Y** | **Y** | **Y** | **Y** | **N** | **N** | **Y** | **8** | **Moderate** |
| Caldas et al. / 2002 | Risk factors associated with asymptomatic infection by *Leishmania chagasi* in northeast Brazil | Previous | **Y** | **Y** | **N** | **Y** | **Y** | **U** | **Y** | **N** | **U** | **U** | **N** | **5** | **Low** |
| Cavalcante / 2009 | Fatores associados à infecção por *Leishmania chagasi* em indivíduos assintomáticos no município de Raposa – MA | Previous | **N** | **Y** | **Y** | **Y** | **Y** | **Y** | **N** | **Y** | **N** | **N** | **N** | **6** | **Moderate** |
| Evans et al. / 1992 | Epidemiology of visceral leishmaniasis in northeast Brazil | Previous | **Y** | **Y** | **Y** | **N** | **N** | **Y** | **Y** | **N** | **N** | **N** | **N** | **5** | **Low** |

**Legend:** **Y= Yes; N= No; U=unclear.** **Q1.** Were the two groups similar and recruited from the same population? **Q2.** Were the exposures measured similarly to assign people to both exposed and unexposed groups? **Q3.** Was the exposure measured in a valid and reliable way? **Q4.** Were confounding factors identified? **Q5.** Were strategies to deal with confounding factors stated? **Q6.** Were the groups/participants free of the outcome at the start of the study (or at the moment of exposure)? **Q7.** Were the outcomes measured in a valid and reliable way? **Q8.** Was the follow up time reported and sufficient to be long enough for outcomes to occur? **Q9.** Was follow up complete, and if not, were the reasons to loss to follow up described and explored? **Q10.** Were strategies to address incomplete follow up utilized? **Q11.** Was appropriate statistical analysis used?

**Quality**:

“Low”: up to five “Yes” responses

“Moderate”: six to nine “Yes” responses

“High”: ten or eleven “Yes” responses

**Case control studies**

| **First Author/ Year** | **Title** | **Review** | **Q1** | **Q2** | **Q3** | **Q4** | **Q5** | **Q6** | **Q7** | **Q8** | **Q9** | **Q10** | **Total** | **Quality** |
| --- | --- | --- | --- | --- | --- | --- | --- | --- | --- | --- | --- | --- | --- | --- |
| Luz et al. **/** 2019 | Are backyard characteristics relevant factors for the occurrence of human visceral leishmaniasis in Central-Western Brazil? | Current | **Y** | **Y** | **Y** | **Y** | **Y** | **Y** | **Y** | **Y** | **Y** | **Y** | **10** | **High** |
| López et al. **/** 2016 | Factores de riesgo, representaciones y prácticas asociadas con la leishmaniasis visceral humana en un foco urbano emergente en Posadas, Argentina | Current | **Y** | **Y** | **N** | **Y** | **Y** | **N** | **N** | **Y** | **Y** | **N** | **6** | **Moderate** |
| Silva et al. **/** 2013 | The Risk Factors for and Effects of Visceral Leishmaniasis in Graft and Renal Transplant Recipients | Current | **Y** | **Y** | **Y** | **Y** | **Y** | **Y** | **Y** | **Y** | **Y** | **U** | **9** | **High** |
| Borges et al. **/** 2008 | Assessment of knowledge and preventive attitudes concerning visceral leishmaniasis in Belo Horizonte, Minas Gerais State, Brazil | Previous | **N** | **Y** | **N** | **N** | **Y** | **Y** | **N** | **N** | **Y** | **N** | **4** | **Low** |
| Borges et al. **/** 2009 | Presença de animais associada ao risco de transmissão da leishmaniose visceral em humanos em Belo Horizonte, Minas Gerais | Previous | **N** | **N** | **N** | **N** | **Y** | **Y** | **N** | **Y** | **Y** | **N** | **4** | **Low** |
| Cabral / 2007 | Influência de fatores ambientais na leishmaniose visceral no Rio Grande do Norte | Previous | **Y** | **N** | **N** | **N** | **Y** | **N** | **N** | **N** | **Y** | **N** | **3** | **Low** |
| Costa et al. / 1999 | Is the  household dog a risk factor for American visceral leishmaniasis in Brazil? | Previous | **Y** | **Y** | **N** | **Y** | **Y** | **N** | **N** | **N** | **Y** | **N** | **5** | **Moderate** |
| Costa et al. / 2005 | Household structure and urban services: neglected targets in the control of  visceral leishmaniasis | Previous | **Y** | **Y** | **Y** | **Y** | **Y** | **Y** | **Y** | **N** | **Y** | **Y** | **9** | **High** |
| Oliveira / 2006 | A case-control study of microenvironmental risk factors for urban visceral leishmaniasis in a large city in Brazil, 1999–2000 | Previous | **Y** | **Y** | **Y** | **N** | **Y** | **Y** | **Y** | **Y** | **Y** | **Y** | **9** | **High** |
| Navin et al. / 1985 | Epidemiologic study of visceral leishmaniasis in Honduras, 1975–1983 | Previous | **Y** | **N** | **N** | **N** | **Y** | **N** | **N** | **N** | **N** | **N** | **2** | **Low** |

**Legend: Y= Yes; N= No; U= Unclear; Q1.** Were the groups comparable other than the presence of disease in cases or the absence of disease in controls? **Q2.** Were cases and controls matched appropriately? **Q3.** Were the same criteria used for identification of cases and controls? **Q4.** Was exposure measured in a standard, valid and reliable way? **Q5.** Was exposure measured in the same way for cases and controls? **Q6.** Were confounding factors identified? **Q7.** Were strategies to deal with confounding factors stated? **Q8.** Were outcomes assessed in a standard, valid and reliable way for cases and controls? **Q9.** Was the exposure period of interest long enough to be meaningful? **Q10.** Was appropriate statistical analysis used?

**Quality:**

“Low”: up to four “Yes” responses

“Moderate”: five to eight “Yes”

“High”: nine or ten “Yes” responses

**Cross-Sectional Studies**

| **First Author/ Year** | **Title** | **Review** | **Q1** | **Q2** | **Q3** | **Q4** | **Q5** | **Q6** | **Q7** | **Q8** | **Total** | **Quality** |
| --- | --- | --- | --- | --- | --- | --- | --- | --- | --- | --- | --- | --- |
| Pastor Santiago, et al. **/** 2012 | American Visceral Leishmaniasis in Chiapas, Mexico | Current | **Y** | **N** | **Y** | **Y** | **Y** | **Y** | **Y** | **Y** | **7** | **High** |
| França, et al. **/** 2013 | Anti-Leishmania antibodies in blood donors from the Midwest region of Brazil | Current | **Y** | **N** | **Y** | **Y** | **Y** | **Y** | **Y** | **Y** | **7** | **High** |
| Villas Boas **/** 2011 | Caracterização dos casos notificados da Leishmaniose Visceral Humana em Governador Valadares, Minas Gerais, no período de 2008-2010 | Current | **Y** | **N** | **Y** | **N** | **N** | **N** | **Y** | **N** | **3** | **Low** |
| Gouvêa **/** 2012 | Associação entre o estado nutricioanl e infecção assintomática por Leishmania infantum em moradores de áreas endêmicas para Leishmaniose visceral no municipio de Teresina, Piauí | Current | **N** | **N** | **Y** | **Y** | **Y** | **Y** | **Y** | **Y** | **6** | **Moderate** |
| Moura et al. **/** 2012 | Factors associated with asymptomatic infection in family members and neighbors of patients with visceral leishmaniasis | Current | **Y** | **Y** | **Y** | **Y** | **Y** | **Y** | **Y** | **Y** | **8** | **High** |
| Lima et al. **/** 2012 | *Leishmania infantum chagasi* in Northeastern Brazil: Asymptomatic Infection at the Urban Perimeter | Current | **Y** | **N** | **Y** | **Y** | **Y** | **Y** | **Y** | **Y** | **7** | **High** |
| Carranza-Tamayo et al. **/** 2016 | Are opossums a relevant factor associated with asymptomatic Leishmania infection in the outskirts of the largest Brazilian cities? | Current | **N** | **Y** | **Y** | **Y** | **Y** | **Y** | **Y** | **Y** | **7** | **High** |
| Maia et al. **/** 2016 | Risk Factors Associated with Human Visceral Leishmaniasis in an Urban Area of Bahia, Brazil | Current | **N** | **Y** | **Y** | **Y** | **Y** | **Y** | **Y** | **U** | **6** | **Moderate** |
| Ponte et al. **/** 2011 | Risk factors for *Leishmania chagasi* infection in an endemic area in Raposa, State of Maranhão, Brazil | Current | **N** | **N** | **Y** | **Y** | **Y** | **Y** | **Y** | **Y** | **6** | **Moderate** |
| Marques **/** 2015 | Associação entre a prevalência da infecção e o risco para o adoecimento por *Leishmania (Leishmania) infantum* em áreas endêmicas distintas de Belo Horizonte, Minas Gerais, Brasil | Current | **Y** | **Y** | **Y** | **Y** | **Y** | **Y** | **Y** | **Y** | **8** | **High** |
| Benitez et al. **/** 2018 | Spatial and simultaneous seroepidemiology of anti-*Leishmania spp.* antibodies in dog owners and their dogs from randomly selected households in a major city of southern Brazil | Current | **Y** | **N** | **Y** | **Y** | **Y** | **Y** | **Y** | **U** | **6** | **Moderate** |
| Caldas et al. / 2002 | Risk factors associated with asymptomatic infection by *Leishmania chagasi* in northeast Brazil | Previous | **N** | **N** | **N** | **Y** | **Y** | **Y** | **Y** | **Y** | **5** | **Moderate** |
| Cavalcante / 2009 | Fatores associados à infecção por *Leishmania chagasi* em indivíduos assintomáticos no município de Raposa – MA | Previous | **Y** | **Y** | **Y** | **N** | **Y** | **Y** | **Y** | **N** | **6** | **Moderate** |
| Coelho / 2009 | Prevalência de desnutrição em menores de 10 anos em área endêmica de leishmaniose visceral | Previous | **N** | **Y** | **Y** | **Y** | **N** | **N** | **Y** | **N** | **4** | **Moderate** |
| Crescente et al. / 2009 | A cross-sectional study on the clinical and immunological spectrum of human  *Leishmania (L.) infantum chagasi* infection in the Brazilian Amazon region | Previous | **Y** | **Y** | **Y** | **Y** | **N** | **N** | **Y** | **N** | **5** | **Moderate** |
| Cunha et al. / 1995 | Visceral leishmaniasis in a new ecological niche near a major metropolitan area of Brazil | Previous | **N** | **Y** | **Y** | **Y** | **N** | **N** | **Y** | **N** | **4** | **Moderate** |
| Cunha et al. / 2001 | Retardo do crescimento em crianças com reação intradérmica positiva para leishmaniose:  resultados preliminares | Previous | **Y** | **N** | **N** | **N** | **N** | **N** | **Y** | **N** | **2** | **Low** |
| Delgado et al. / 1998 | The re-emergence of American visceral leishmaniasis in an old focus in Venezuela: present situation of human and canine infections | Previous | **N** | **N** | **N** | **Y** | **N** | **N** | **Y** | **N** | **2** | **Low** |
| D’Oliveira-Júnior et al. / 1997 | Asymptomatic Leishmania chagasi Infection in Relatives and Neighbors of Patients  with Visceral Leishmaniasis | Previous | **Y** | **N** | **Y** | **Y** | **N** | **N** | **Y** | **N** | **4** | **Moderate** |
| Evans et al. / 1992 | Epidemiology of visceral leishmaniasis in northeast Brazil | Previous | **N** | **N** | **Y** | **Y** | **N** | **N** | **Y** | **N** | **3** | **Low** |
| Falqueto / 2009 | Cross-sectional and longitudinal epidemiologic surveys of human and canine  Leishmania infantum visceral infections in an endemic rural area of southeast Brazil | Previous | **Y** | **Y** | **Y** | **Y** | **N** | **N** | **Y** | **N** | **4** | **Moderate** |
| Feliciangeli et al. / 2005 | The burden of the Leishmania chagasi/infantum infection in a closed rural focus of visceral leishmaniasis in Lara state, west-central Venezuela | Previous | **N** | **Y** | **N** | **Y** | **N** | **N** | **Y** | **N** | **3** | **Low** |
| Feliciangeli et al. / 2006 | Leishmania and sand flies: proximity to woodland as a risk factor for infection in a rural focus of visceral leishmaniasis in west central Venezuela | Previous | **N** | **Y** | **N** | **Y** | **N** | **N** | **Y** | **N** | **3** | **Low** |
| Gouvêa / 2007 | Factors associated  to Montenegro skin test positivity in Teresina, Brazil | Previous | **Y** | **Y** | **Y** | **Y** | **Y** | **Y** | **Y** | **Y** | **8** | **High** |
| Jeronimo et al. / 2004 | An  emerging peri-urban pattern of infection with *Leishmania chagasi*, the protozoan  causing visceral leishmaniasis in northeast Brazi | Previous | **N** | **N** | **Y** | **Y** | **N** | **N** | **Y** | **N** | **3** | **Low** |
| Lima / 2010 | Aspectos ambientais e sociais envolvidos na transmissão da *L.Chagasi* no município de Parnamirim/RN | Previous | **N** | **N** | **N** | **Y** | **N** | **N** | **Y** | **N** | **2** | **Low** |
| Moreno et al. / 2005 | Risk factors for Leishmania chagasi infection in an urban area of Minas Gerais  State | Previous | **N** | **N** | **Y** | **Y** | **Y** | **N** | **Y** | **N** | **4** | **Moderate** |
| Nascimento et al. / 2005 | Prevalence of infection by Leishmania chagasi using ELISA (rK39 and CRUDE) and the Montenegro skin test in an endemic leishmaniasis area of Maranhão, Brazil | Previous | **N** | **N** | **N** | **Y** | **N** | **N** | **Y** | **N** | **2** | **Low** |
| Oliveira / 2008 | Asymptomatic infection in family contacts of patients with human visceral leishmaniasis in Três Lagoas, Mato Grosso do Sul State, Brazil | Previous | **Y** | **Y** | **N** | **Y** | **N** | **N** | **Y** | **N** | **4** | **Moderate** |
| Viana et al. / 2008 | Combined diagnostic methods identify a remarkable proportion of asymptomatic *Leishmania (Leishmania) chagasi* carriers who present modulated cytokine  profiles | Previous | **N** | **N** | **Y** | **N** | **N** | **N** | **Y** | **N** | **2** | **Low** |
| Werneck et al. / 2002 | The burden of *Leishmania chagasi* infection during an urban outbreak of visceral leishmaniasis in Brazil | Previous | **Y** | **N** | **Y** | **Y** | **N** | **N** | **Y** | **N** | **4** | **Moderate** |
| Barão et al. / 2007 | Human asymptomatic infection in visceral leishmaniasis: a seroprevalence study in an  urban area of low endemicity | Previous | **N** | **Y** | **N** | **Y** | **N** | **N** | **Y** | **N** | **3** | **Low** |
| Corredor et al. / 1989 | Epidemiology of visceral leishmaniasis in Colombia | Previous | **N** | **N** | **Y** | **Y** | **N** | **N** | **Y** | **N** | **3** | **Low** |
| Corredor et al./ 1999 | Prevalence of Trypanosoma cruzi and Leishmania chagasi infection and risk  factors in a Colombian indigenous population | Previous | **Y** | **Y** | **Y** | **Y** | **N** | **N** | **Y** | **N** | **5** | **Moderate** |
| Luz et al. / 1997 | Prevalence of anti-Leishmania donovani antibody among Brazilian blood donors and multiply transfused hemodialysis patient | Previous | **N** | **N** | **Y** | **Y** | **N** | **N** | **Y** | **N** | **3** | **Low** |

**Legend: Y= Yes; N= No; U= Unclear; NA= Not applicable; Q1.** Were the criteria for inclusion in the sample clearly defined? **Q2.** Were the study subjects and the setting described in detail? **Q3.** Was the exposure measured in a valid and reliable way? **Q4.** Were objective, standard criteria used for measurement of the condition? **Q5.** Were confounding factors identified? **Q6.** Were strategies to deal with confounding factors stated? **Q7.** Were the outcomes measured in a valid and reliable way? **Q8.** Was appropriate statistical analysis used?

**Quality:**

“Low”: up to three “Yes” responses

“Moderate”: four to six “Yes”

“High”: seven or eight “Yes” responses
