# Supplementary material for: An updated systematic review with meta-analysis and meta-regression of the factors associated with human visceral leishmaniasis in the Americas
Source: Infect Dis Poverty. 2025 Jan 30;14:4. doi: 10.1186/s40249-025-01274-z (PMC11781006; doi:10.1186/s40249-025-01274-z)
Supplement: Supplementary file 4 — Additional file 4. Description of Q values, P values and I². [file 40249_2025_1274_MOESM4_ESM.docx]

**Additional file 4: Description of Q values, *P* values ​​and *I*²**

| **Variable/moderators** | **Q-value** | ***P*-value** | ***I*-squared (*I*²)** |
| --- | --- | --- | --- |
| **Sex** | 75,540 | 0,000 | 54,991 |
| Confounding control/No | 39,942 | 0,005 | 49,927 |
| Confounding control/Yes | 16,806 | 0,032 | 52,397 |
| Confounding control/Total within | 56,747 | 0,001 | - |
| Confounding control/Total between | 9,897 | 0,002 | - |
| **Age (≥ 10 vs < 10 years)** | 149,916 | 0,000 | 87,326 |
| Measurement of the outcome/Clinical cases | 2,755 | 0,097 | 63,706 |
| Measurement of the outcome/LST | 40,223 | 0,000 | 72,652 |
| Measurement of the outcome/LST and Serological | 0,000 | 1,000 | 0,000 |
| Measurement of the outcome Other | 1,390 | 0,499 | 0,000 |
| Measurement of the outcome/Serological | 8,644 | 0,124 | 42,159 |
| Measurement of the outcome/Total within | 53,013 | 0,000 | - |
| Measurement of the outcome/Total between | 106,444 | 0,000 | - |
| **Age (elderly and close-ups vs children < 10)** | 26,368 | 0,001 | 69,661 |
| Presence of dogs in the domicile | - | 0,005 | 50,265 |
| Type of study/Case control | 11,626 | 0,040 | 56,994 |
| Type of study/Cohort | 0,801 | 0,849 | 0,000 |
| Type of study/CS | 12,319 | 0,264 | 18,823 |
| Type of study/Total within | 24,746 | 0,132 | - |
| Type of study/Total between | 15,467 | 0,000 | - |
| **Presence of a seropositive dog in the domicile** | 16,769 | 0,001 | 82,110 |
| **Presence of chickens/other fowl at the domicile** | 39,677 | 0,001 | 57,154 |
| Type of study/Case control | 0,571 | 0,752 | 0,000 |
| Type of study/Cohort | 2,740 | 0,433 | 0,000 |
| Type of study/CS | 29,683 | 0,001 | 66,310 |
| Type of study/Total within | 32,994 | 0,005 | - |
| Type of study/Total between | 6,683 | 0,035 | - |
| Confounding control/No | 7,501 | 0,585 | 0,000 |
| Confounding control/Yes | 26,669 | 0,000 | 73,753 |
| Confounding control/Total within | 34,171 | 0,005 | - |
| Confounding control/Total between | 5,507 | 0,019 | - |
| **Presence of pigs or pigsty** | 3,422 | 0,635 | 0,000 |
| **Presence of cat** | 5,594 | 0,232 | 28,490 |
| **Prior contact with infected household member, relatives or neighbors** | - | - | - |
| Neighborhood | 0,621 | 0,892 | 0,000 |
| Relatives | 20,777 | 0,008 | 61,495 |
| Total within | 21,397 | 0,029 | - |
| Total between | 11,975 | 0,001 | - |
| Confounding control/No | 24,942 | 0,000 | 75,944 |
| Confounding control/Yes | 7,285 | 0,200 | 31,365 |
| Confounding control/Total within | 32,227 | 0,001 | - |
| Confounding control/Total between | 1,145 | 0,285 | - |
| **Water supply** | 23,190 | 0,001 | 74,127 |
| **Garbage disposal** | 28,858 | 0,000 | 80,664 |
| **Sewage collection** | 25,055 | 0,000 | 80,044 |
| **Income** | 0,565 | 0,754 | 0,000 |
| **Education (illiterate vs some education)** | 9,634 | 0,022 | 68,860 |
| **Education (elementary and non-elementary)** | 13,362 | 0,004 | 77,548 |
| **Floor** | 42,742 | 0,000 | 81,283 |
| **House finishing** | 38,325 | 0,000 | 73,907 |
| **House walls** | 7,502 | 0,112 | 46,682 |
| **Number of people per household (≥ 4 people vs < 4)** | 1,497 | 0,827 | 0,000 |
| **Number of people per room** | 10,597 | 0,031 | 62,254 |
| **Accessible backyard at the domicile or nearby** | 2,962 | 0,814 | 0,000 |
