# Supplementary material for: An updated systematic review with meta-analysis and meta-regression of the factors associated with human visceral leishmaniasis in the Americas
Source: Infect Dis Poverty. 2025 Jan 30;14:4. doi: 10.1186/s40249-025-01274-z (PMC11781006; doi:10.1186/s40249-025-01274-z)
Supplement: Supplementary file 5 — Additional file 5. Forest plots for variables with subgroup analyses by controlling for confounding. Fig. S1 Forest plot for the sex variable: studies divided into subgroups by confounding control. Abbreviations: CS cross-sectional; LST Leishmania skin test. Superscripts:result of serological test in a study involving two diagnostic tests;results in adults;second result in a single article. The squares represent the weight of each study, whereas the diamonds represent the summary measurement of each subgroup. Reference: Female, Odds ratio = 1. [14, 15, 33, 36, 37, 39–41, 42, 43, 45, 46, 48 – 51, 53, 55–58, 60, 63, 65, 66, 68, 69]. Fig. S2 Forest plot for the age variable: studies divided into subgroups by confounding control. Abbreviations: CS cross-sectional; LST Leishmania skin test. Superscripts:result of serological test in a study involving two diagnostic tests;results in adults;second result in a single article. The squares represent the weight of each study, whereas the diamonds represent the summary measurement of each subgroup. Reference: Being over 10 years old, Odds Ratio = 1. [14, 15, 28, 30, 38–40, 42, 48–50, 53, 55–60, 63]. Fig. S3 Forest plot for the variable presence of dog in the domicile: studies divided into subgroups by confounding control. Abbreviations: CS cross-sectional; LST Leishmania skin test. Superscripts:result of serological test in a study involving two diagnostic tests;results in adults;second result in a single article. The squares represent the weight of each study, whereas the diamonds represent the summary measurement of each subgroup. Reference: Not having dogs, Odds Ratio = 1. [15, 27, 35, 37, 39, 42, 43, 48, 52, 53, 58–60, 63, 65, 67–68]. Fig. S4 Forest plot for the variable presence of chickens/other fowl at the domicile: studies divided into subgroups by confounding control. Abbreviations: CS cross-sectional; LST Leishmania skin test. Superscripts:result of serological test in a study involving two diagnostic tests;results in [file 40249_2025_1274_MOESM5_ESM.docx]

**Additional file 5: Forest plots for variables with subgroup analyses by controlling for confounding**


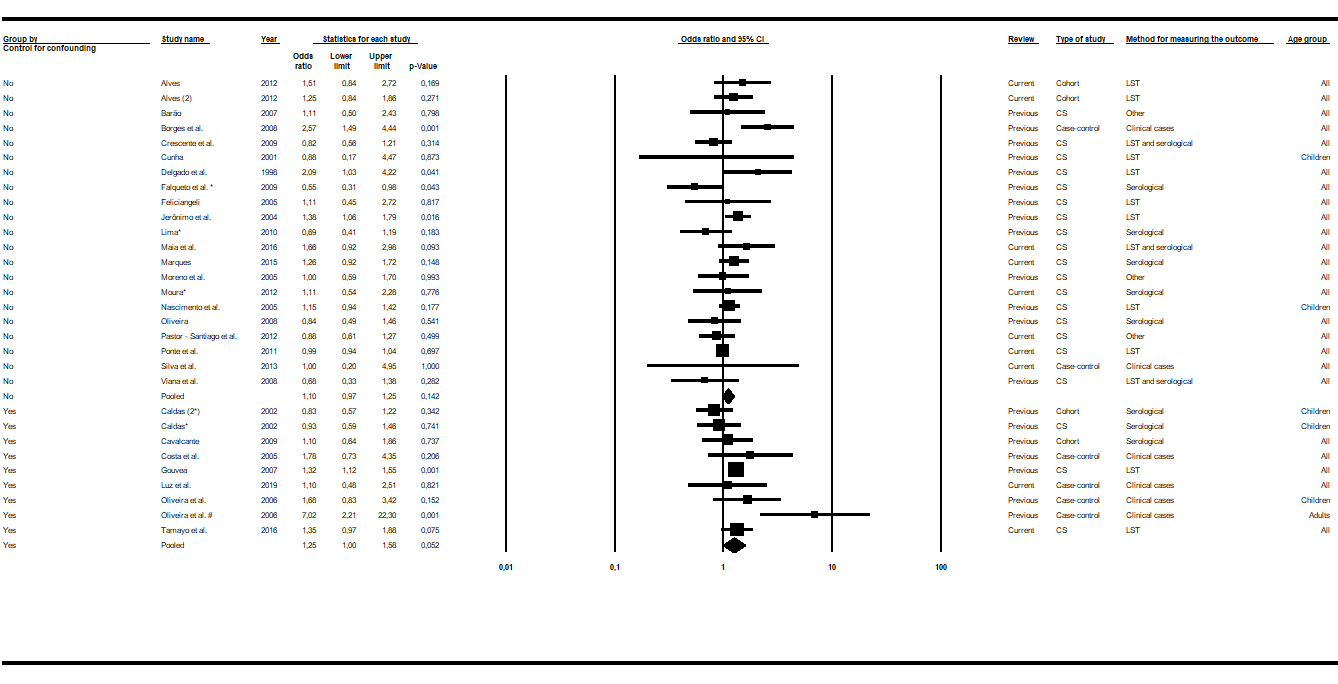


**Fig. S1** Forest plot for the sex variable: studies divided into subgroups by confounding control. Abbreviations: *CS* cross-sectional; *LST* *Leishmania* skin test. Superscripts: (*) result of serological test in a study involving two diagnostic tests; (#) results in adults; (2) second result in a single article. The squares represent the weight of each study, whereas the diamonds represent the summary measurement of each subgroup. Reference: Female, Odds ratio = 1. [14, 15, 33, 36, 37, 39–41, 42, 43, 45, 46, 48 – 51, 53, 55–58, 60, 63, 65, 66, 68, 69]


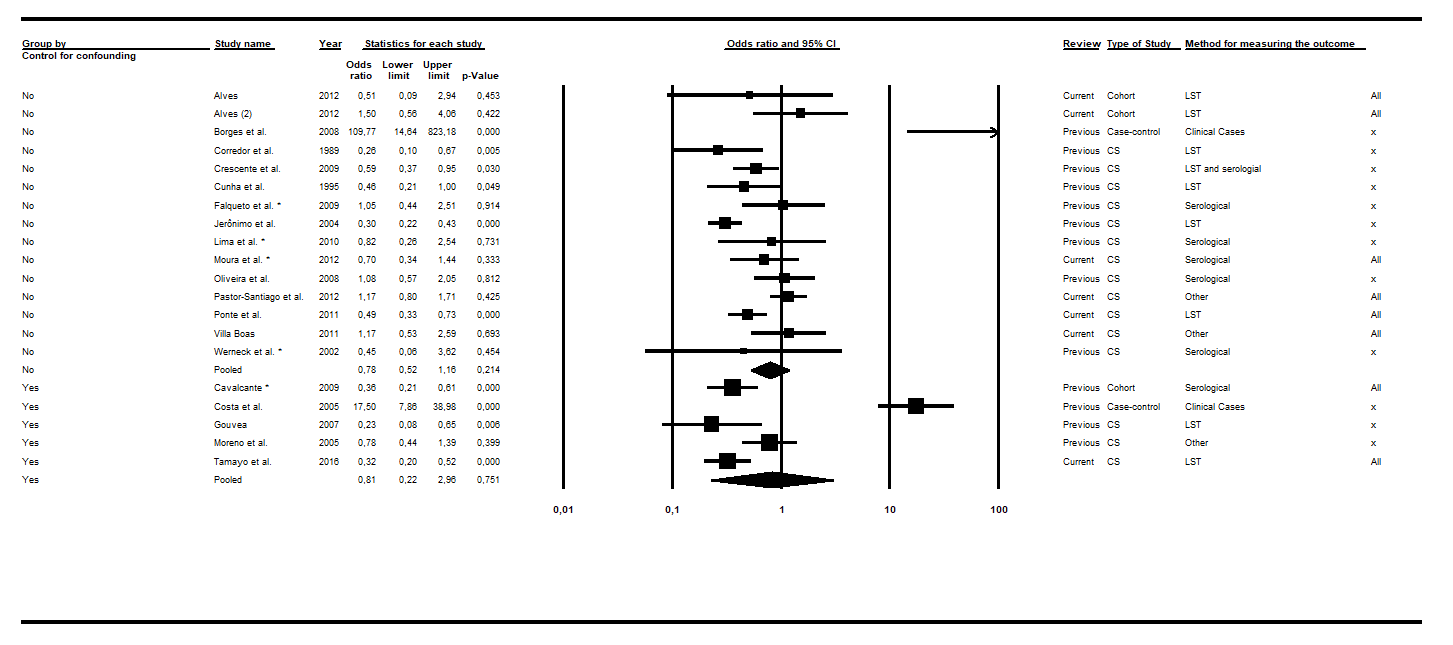


**Fig. S2** Forest plot for the age variable: studies divided into subgroups by confounding control. Abbreviations: *CS* cross-sectional; *LST* *Leishmania* skin test. Superscripts: (*) result of serological test in a study involving two diagnostic tests; (#) results in adults; (2) second result in a single article. The squares represent the weight of each study, whereas the diamonds represent the summary measurement of each subgroup. Reference: Being over 10 years old, Odds Ratio = 1. [14, 15, 28, 30, 38–40, 42, 48–50, 53, 55–60, 63]


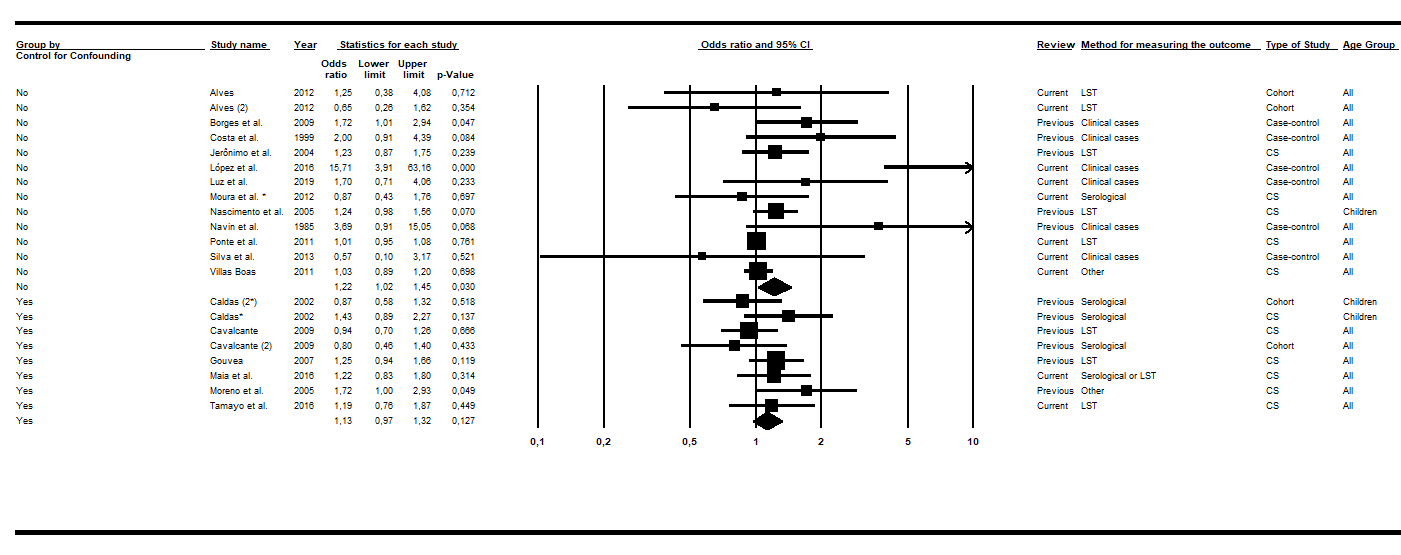


**Fig. S3** Forest plot for the variable presence of dog in the domicile: studies divided into subgroups by confounding control. Abbreviations: *CS* cross-sectional; *LST* *Leishmania* skin test. Superscripts: (*) result of serological test in a study involving two diagnostic tests; (#) results in adults; (2) second result in a single article. The squares represent the weight of each study, whereas the diamonds represent the summary measurement of each subgroup. Reference: Not having dogs, Odds Ratio = 1. [15, 27, 35, 37, 39, 42, 43, 48, 52, 53, 58–60, 63, 65, 67–68]


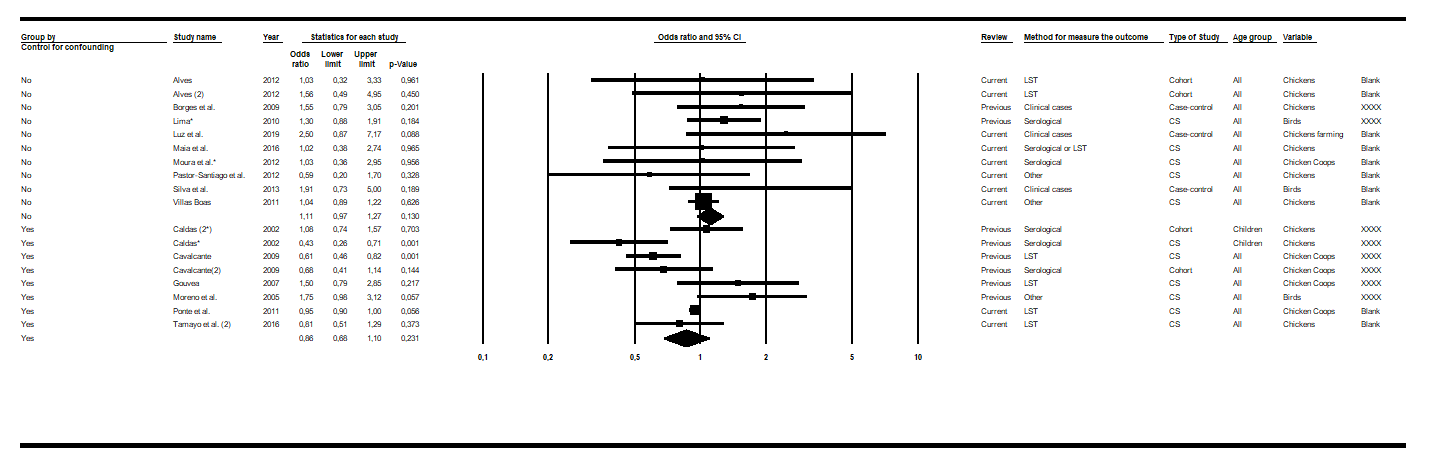


**Fig. S4** Forest plot for the variable presence of chickens/other fowl at the domicile: studies divided into subgroups by confounding control. Abbreviations: *CS* cross-sectional; *LST Leishmania* skin test. Superscripts: (*) result of serological test in a study involving two diagnostic tests; (#) results in adults; (2) second result in a single article. The squares represent the weight of each study, whereas the diamonds represent the summary measurement of each subgroup. Reference: Do not have chickens and poultry, Odds Ratio = 1. [14, 15, 37, 42, 48, 52, 53, 57 – 60, 63, 65, 68, 69]


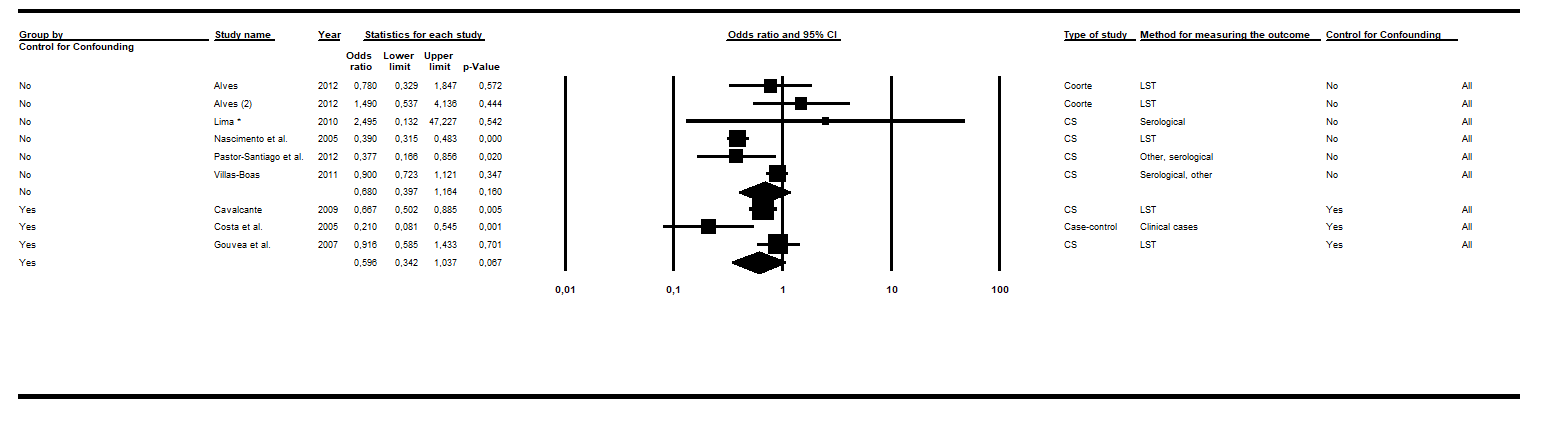


**Fig. S5** Forest plot for the floor variable: studies divided into subgroups by confounding control. Abbreviations: *CS* cross-sectional; *LST* *Leishmania* skin test. Superscripts: (*) result of serological test in a study involving two diagnostic tests; (#) results in adults; (2) second result in a single article. The squares represent the weight of each study, whereas the diamonds represent the summary measurement of each subgroup. Reference: Inadequate, Odds Ratio = 1. [14, 40, 43, 48, 53, 57, 59, 60]


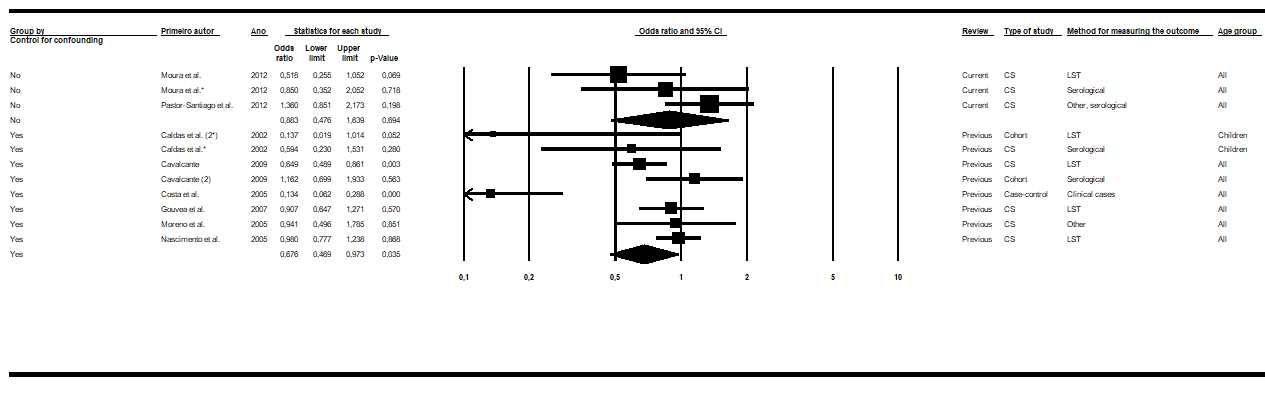


**Fig. S6** Forest plot for the variable house finishing: studies divided into subgroups by confounding control. Abbreviations: *CS* cross-sectional; *LST* *Leishmania* skin test. Superscripts: (*) result of serological test in a study involving two diagnostic tests; (#) results in adults; (2) second result in a single article. The squares represent the weight of each study, whereas the diamonds represent the summary measurement of each subgroup. Reference: Inadequate, Odds Ratio = 1. [14, 37, 40, 42, 43, 48, 53, 63]
