# Supplementary material for: An updated systematic review with meta-analysis and meta-regression of the factors associated with human visceral leishmaniasis in the Americas
Source: Infect Dis Poverty. 2025 Jan 30;14:4. doi: 10.1186/s40249-025-01274-z (PMC11781006; doi:10.1186/s40249-025-01274-z)

**Additional file 6:** Forest plot for the variable presence of other animals at the domicile: (**a)** cattle/horses; **(b)** cats; **(c)** pigs**.** Abbreviations: *CS*,cross-sectional; *LST* *Leishmania* skin test. Superscripts: (*) result of serological test in a study involving two diagnostic tests; (#) results in adults; (2) second result in a single article. The squares represent the weight of each study, whereas the diamonds represent the summary measurement of each subgroup. Category of reference: absence of pigs, cats and cattle/horses, Odds ratio = 1. References: [14, 15, 58, 59, 63, 65, 68, 69]


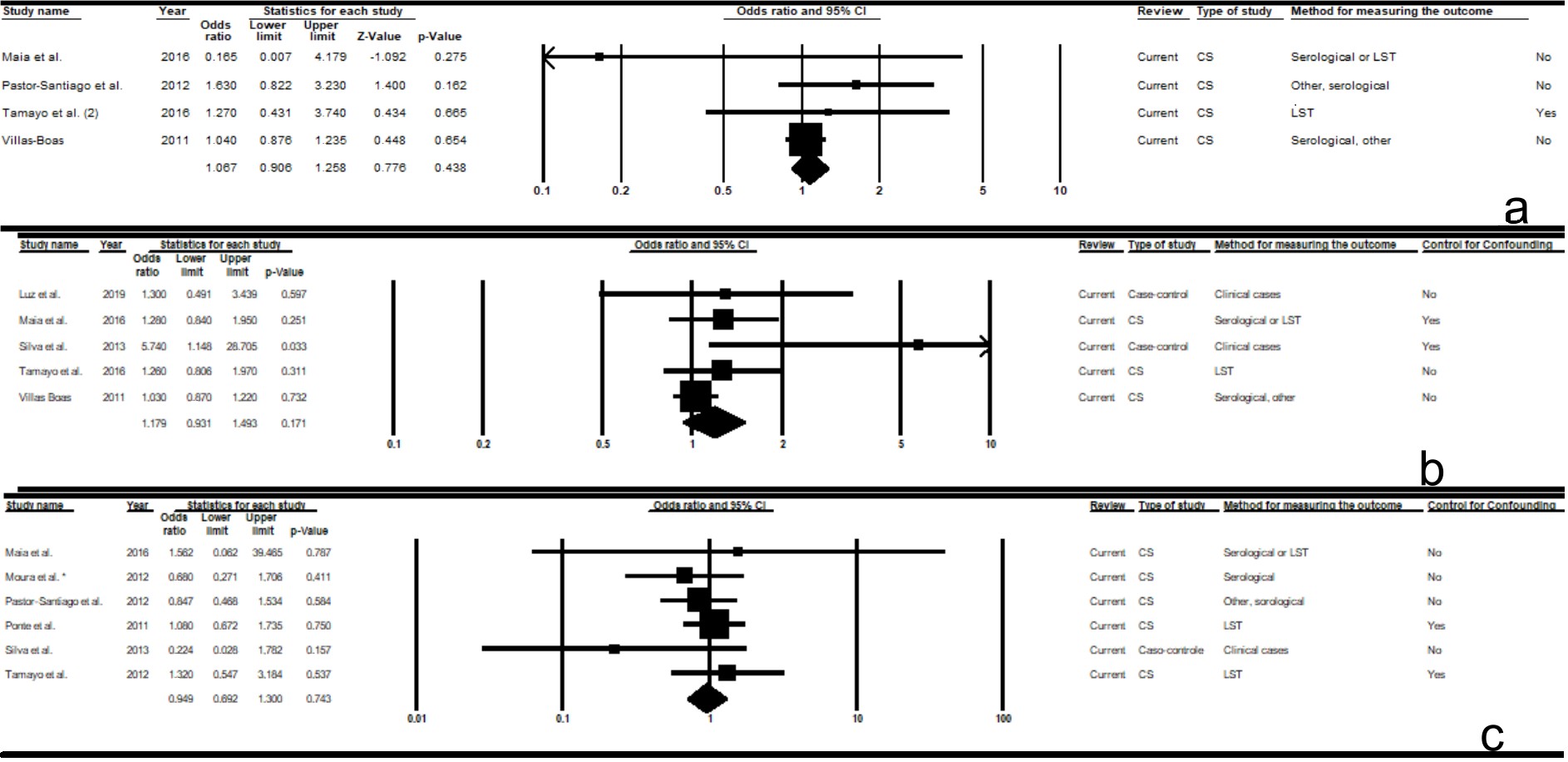

Supplement: Supplementary file 6 — Additional file 6. Forest plot for the variable presence of other animals at the domicile:cattle/horses;cats;pigs. Abbreviations: CS,cross-sectional; LST Leishmania skin test. Superscripts:result of serological test in a study involving two diagnostic tests;results in adults;second result in a single article. The squares represent the weight of each study, whereas the diamonds represent the summary measurement of each subgroup. Category of reference: absence of pigs, cats and cattle/horses, Odds ratio = 1. References: [14, 15, 58, 59, 63, 65, 68, 69]. [file 40249_2025_1274_MOESM6_ESM.docx]
