# Supplementary material for: An updated systematic review with meta-analysis and meta-regression of the factors associated with human visceral leishmaniasis in the Americas
Source: Infect Dis Poverty. 2025 Jan 30;14:4. doi: 10.1186/s40249-025-01274-z (PMC11781006; doi:10.1186/s40249-025-01274-z)
Supplement: Supplementary file 7 — Additional file 7. Forest plots of socioeconomic variables associated with human visceral leishmaniasis in the Americas. Fig. S1 Forest plot for the water supply variable. Abbreviations: CS cross-sectional; LST Leishmania skin test. Superscripts:result of serological test in a study involving two diagnostic tests;results in adults;second result in a single article. The squares represent the weight of each study, whereas the diamonds represent the summary measurement of each subgroup. Reference: Non-piped water, Odds Ratio = 1. [40, 43, 48, 60, 63, 65]. Fig. S2 Forest plot for the sewage system variable. Abbreviations: CS cross-sectional; LST Leishmania skin test. Superscripts:result of serological test in a study involving two diagnostic tests;results in adults;second result in a single article. The squares represent the weight of each study, whereas the diamonds represent the summary measurement of each subgroup. Reference: Absence of sewage system, Odds Ratio = 1. [40, 48, 53, 63, 69]. Fig. S3 Forest plot for the garbage collection variable. Abbreviations: CS cross-sectional; LST Leishmania skin test. Superscripts:result of serological test in a study involving two diagnostic tests;results in adults;second result in a single article. The squares represent the weight of each study, whereas the diamonds represent the summary measurement of each subgroup. Reference: Public garbage collection, Odds Ratio = 1. [40, 42, 53, 63, 65, 67]. Fig. S4 Forest plot for the floor variable. Abbreviations: CS cross-sectional; LST Leishmania skin test. Superscripts:result of serological test in a study involving two diagnostic tests;results in adults;second result in a single article. The squares represent the weight of each study, whereas the diamonds represent the summary measurement of each subgroup. Reference: Inadequate, Odds Ratio = 1. [14, 40, 43, 48, 53, 57, 59, 60]. Fig. S5 Forest plot for the house finishing variable. Abbreviations: CS cross-sectional; LST Leishmania skin [file 40249_2025_1274_MOESM7_ESM.docx]

**Additional file 7: Forest Plots of Socioeconomic Variables Associated with Human Visceral Leishmaniasis in the Americas**


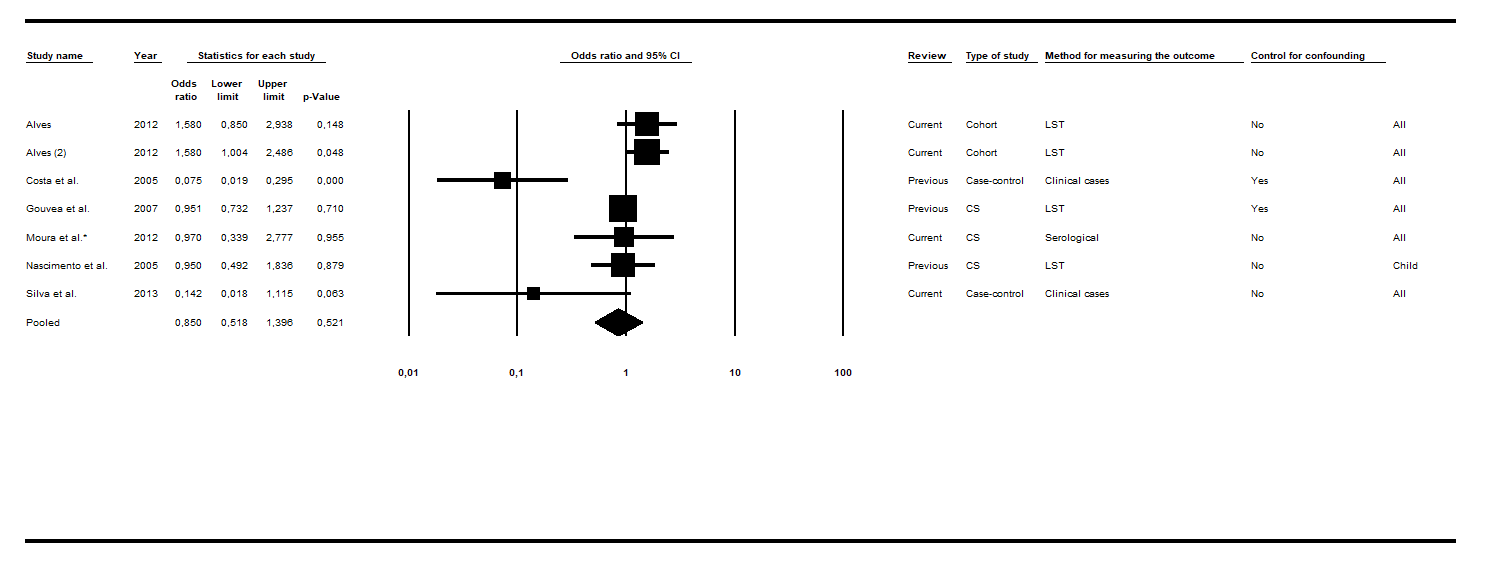


**Fig. S1** Forest plot for the water supply variable. Abbreviations: *CS* cross-sectional; *LST* *Leishmania* skin test. Superscripts: (*) result of serological test in a study involving two diagnostic tests; (#) results in adults; (2) second result in a single article. The squares represent the weight of each study, whereas the diamonds represent the summary measurement of each subgroup. Reference: Non-piped water, Odds Ratio = 1. [40, 43, 48, 60, 63, 65]


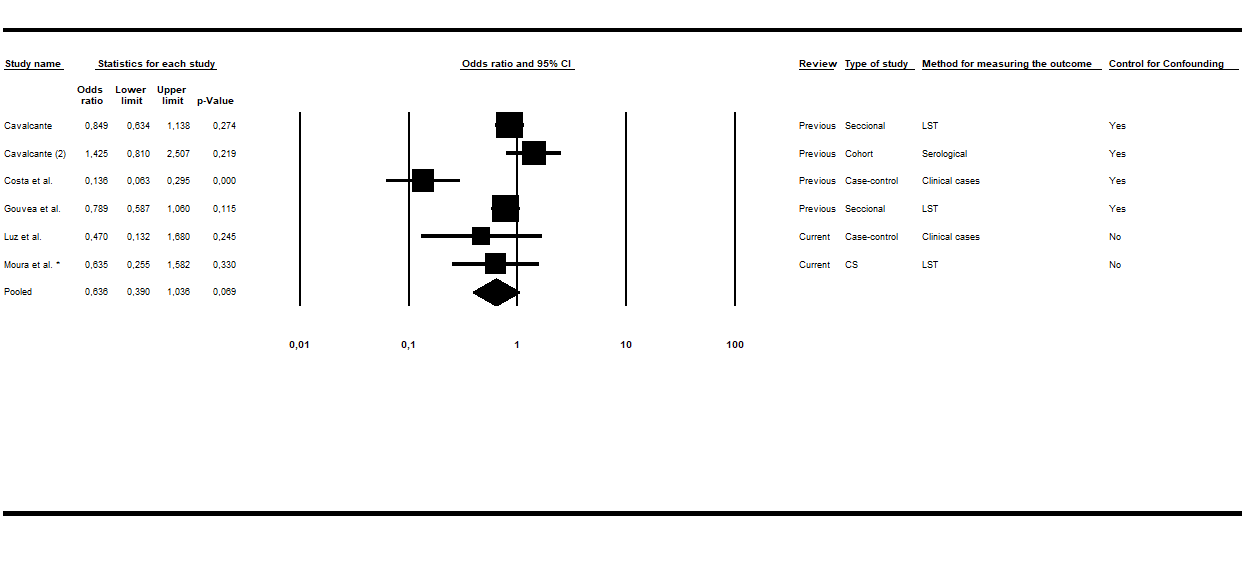
 **Fig. S2** Forest plot for the sewage system variable. Abbreviations: *CS* cross-sectional; *LST* *Leishmania* skin test. Superscripts: (*) result of serological test in a study involving two diagnostic tests; (#) results in adults; (2) second result in a single article. The squares represent the weight of each study, whereas the diamonds represent the summary measurement of each subgroup. Reference: Absence of sewage system, Odds Ratio = 1. [40, 48, 53, 63, 69]


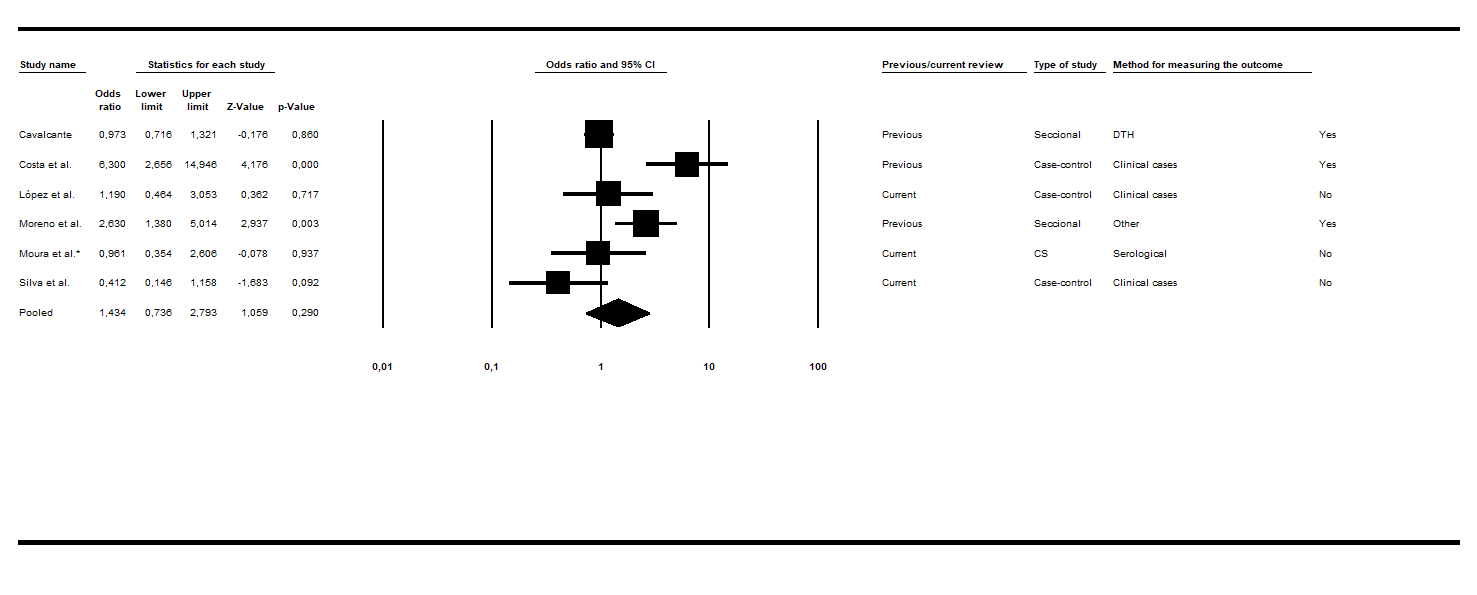


**Fig. S3** Forest plot for the garbage collection variable. Abbreviations: *CS* cross-sectional; *LST* *Leishmania* skin test. Superscripts: (*) result of serological test in a study involving two diagnostic tests; (#) results in adults; (2) second result in a single article. The squares represent the weight of each study, whereas the diamonds represent the summary measurement of each subgroup. Reference: Public garbage collection, Odds Ratio = 1. [40, 42, 53, 63, 65, 67]


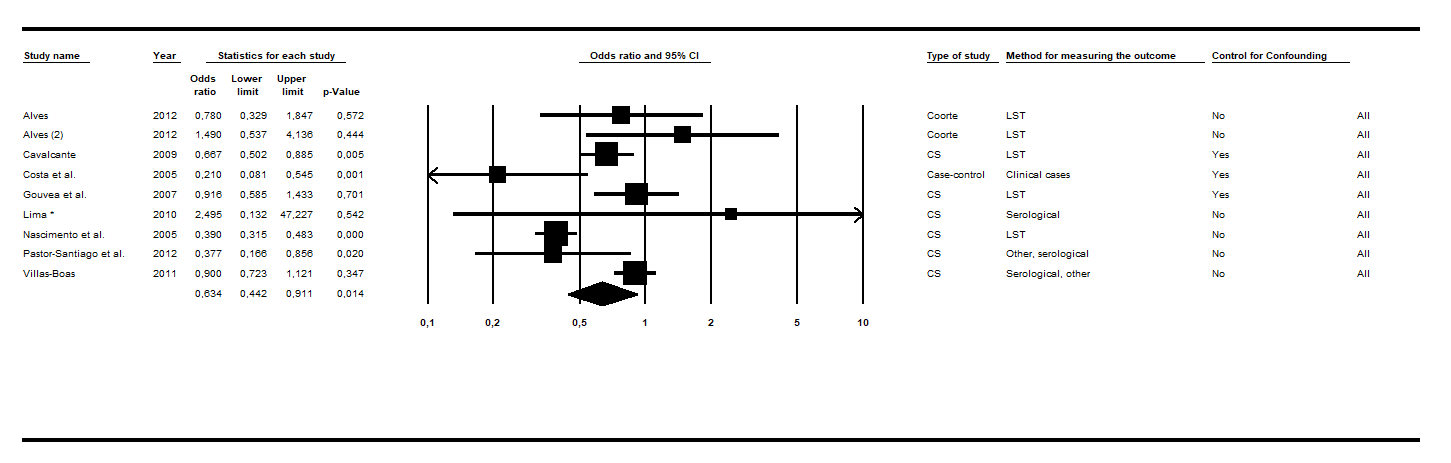


**Fig. S4** Forest plot for the floor variable. Abbreviations: *CS* cross-sectional; *LST* *Leishmania* skin test. Superscripts: (*) result of serological test in a study involving two diagnostic tests; (#) results in adults; (2) second result in a single article. The squares represent the weight of each study, whereas the diamonds represent the summary measurement of each subgroup. Reference: Inadequate, Odds Ratio = 1. [14, 40, 43, 48, 53, 57, 59, 60]


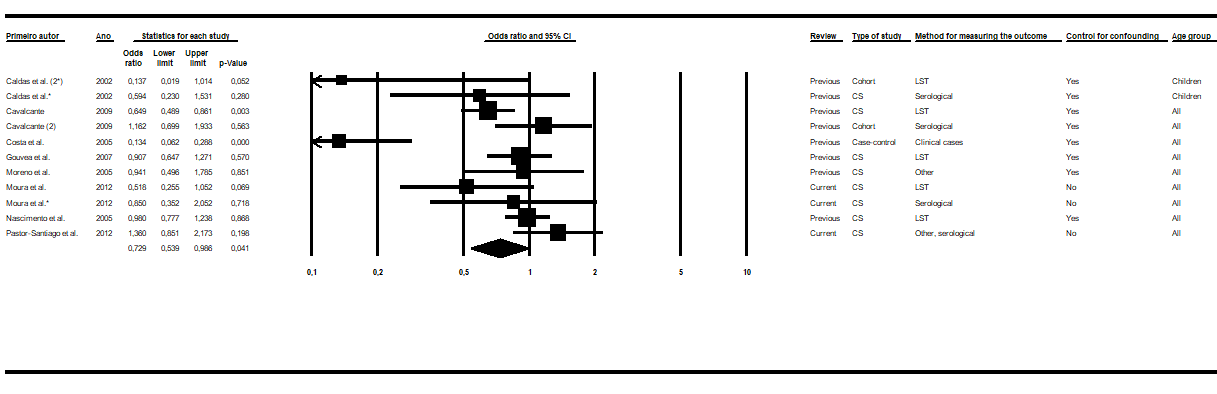


**Fig. S5** Forest plot for the house finishing variable. Abbreviations: *CS* cross-sectional; *LST* *Leishmania* skin test. Superscripts: (*) result of serological test in a study involving two diagnostic tests; (#) results in adults; (2) second result in a single article. The squares represent the weight of each study, whereas the diamonds represent the summary measurement of each subgroup. Reference: Inadequate, Odds Ratio = 1. [14, 37, 40, 42, 43, 48, 53, 63]


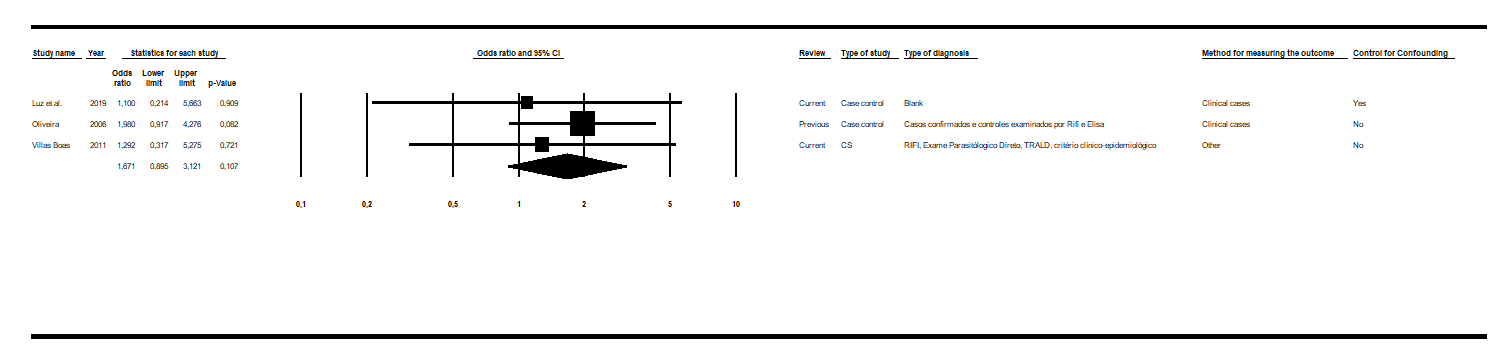


**Fig. S6** Forest plot for the income variable. Abbreviations: *CS* cross-sectional; *LST* *Leishmania* skin test. Superscripts: (*) result of serological test in a study involving two diagnostic tests; (#) results in adults; (2) second result in a single article. The squares represent the weight of each study, whereas the diamonds represent the summary measurement of each subgroup. Reference: More than 01 minimum wage, Odds Ratio = 1. [45, 59, 69]


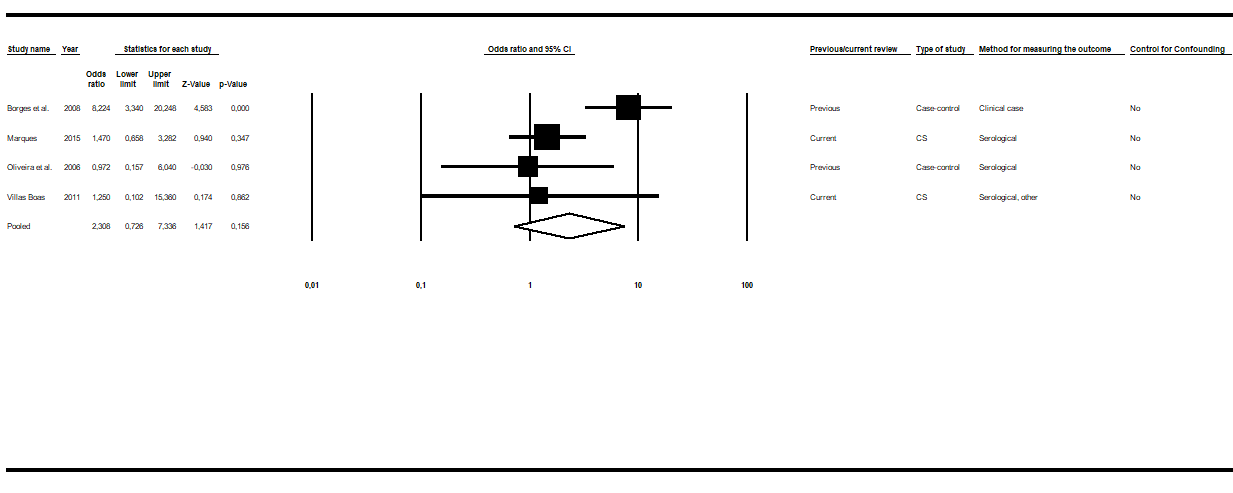
 **Fig. S7** Forest plot for the variable education. Abbreviations: *CS* cross-sectional; *LST* *Leishmania* skin test. Superscripts: (*) result of serological test in a study involving two diagnostic tests; (#) results in adults; (2) second result in a single article. The squares represent the weight of each study, whereas the diamonds represent the summary measurement of each subgroup. Reference: Some education, Odds Ratio = 1. [45, 49, 59, 66]


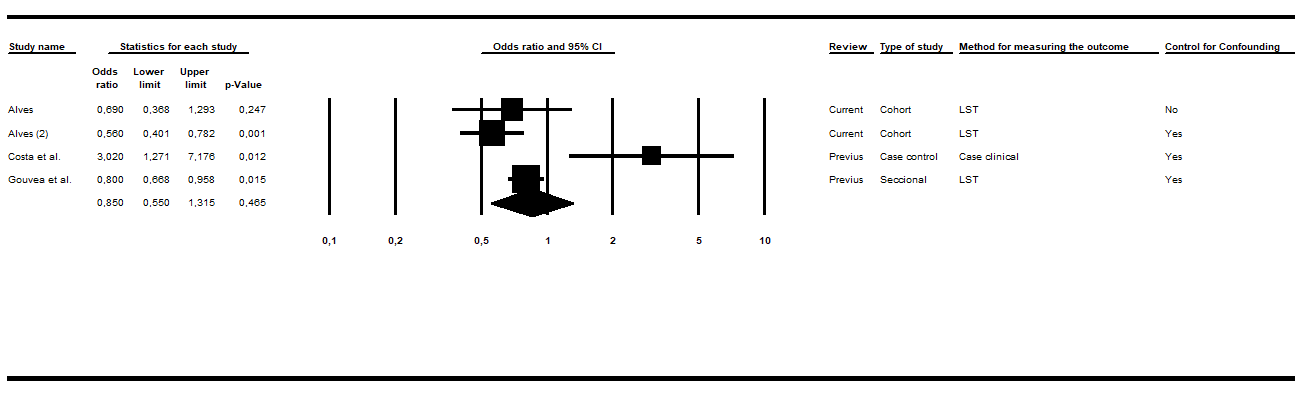


**Fig. S8**Forest plot for the variable education. Abbreviations: *CS* cross-sectional; *LST* *Leishmania* skin test. Superscripts: (*) result of serological test in a study involving two diagnostic tests; (#) results in adults; (2) second result in a single article. The squares represent the weight of each study, whereas the diamonds represent the summary measurement of each subgroup. Reference: Non-elementary, Odds Ratio = 1. [40, 48, 60]


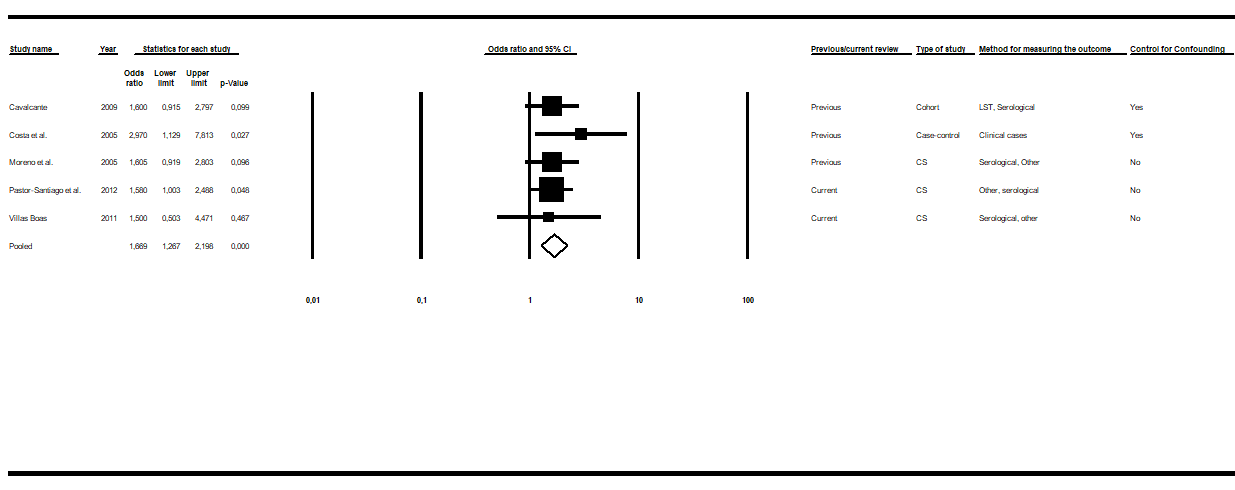


**Fig. 9** Forest plot for the variable number of residents per household. Abbreviations: *CS* cross-sectional; *LST* *Leishmania* skin test. Superscripts: (*) result of serological test in a study involving two diagnostic tests; (#) results in adults; (2) second result in a single article. The squares represent the weight of each study, whereas the diamonds represent the summary measurement of each subgroup. Reference: < 4 people, Odds Ratio = 1. [14, 40, 42, 53, 59]
