# Supplementary material for: An updated systematic review with meta-analysis and meta-regression of the factors associated with human visceral leishmaniasis in the Americas
Source: Infect Dis Poverty. 2025 Jan 30;14:4. doi: 10.1186/s40249-025-01274-z (PMC11781006; doi:10.1186/s40249-025-01274-z)
Supplement: Supplementary file 8 — Additional file 8. Results of analyses and interpretation of the level of evidence using the GRADE approach. Table S1. Interpretation of the quality of evidence according to the GRADE methodology. Table S2. Results of the GRADE approach by domains [file 40249_2025_1274_MOESM8_ESM.docx]

**Additional file 8: Results of analyses and interpretation of the level of evidence using the GRADE approach**

**Table S1.** Interpretation of the quality of evidence according to the GRADE methodology

| **Quality level** | **Interpretation** |
| --- | --- |
| **High certainty** | We are very confident that the true effect is close to the effect estimate. |
| **Moderate certainty** | The true effect is probably close to the effect estimate, but there is a possibility that it is substantially different. |
| **Low certainty** | The true effect may be substantially different from the estimate of the effect. |
| **Very low certainty** | The true effect is likely to be substantially different from the estimate of the effect. |

**Table S2.** Results of the GRADE approach by domains

| **Risk fator** | **No of studies included** | **Study design** | **Risk of bias** | **Inconsistency** | **Indirectness** | **Imprecision** | **Publication bias** | **Quality of evidence** | **Comments** |
| --- | --- | --- | --- | --- | --- | --- | --- | --- | --- |
| Sex | 27 | Observational | Not serious^a b^ | Not serious^g^ | Not serious^i^ | Not serious^j^ | Not serious^l^ | ⊕⊕⊕◯  MODERATE | Evidence raised to one level due to the magnitude of the effect, with clinical relevance (greater chance of symptomatic cases in males) |
| Age (comparison between individuals older or younger than 10 years old) | 19 | Observational | Not serious^a b^ | Not serious^g^ | Not serious^i^ | Not serious^j^ | Not serious^l^ | ⊕⊕⊕◯  MODERATE | Evidence raised to one level due to the magnitude of the effect, with clinical relevance (greater chance of symptomatic cases in children) |
| Age (comparison between individuals under 10 and over 50 years old) | 8 | Observational | Serious^a c^ | Serious^f^ | Not serious^i^ | Serious^k^ | Not assessed^n^ | ⊕◯◯◯ VERY LOW | Absence of factors that increase the quality of evidence |
| Presence of dogs in the domicile | 21 | Observational | Serious^a^ ^c^ | Not serious^g^ | Not serious^i^ | Not serious^j^ | Serious^m^ | ⊕◯◯◯ VERY LOW | Absence of factors that increase the quality of evidence |
| Presence of a seropositive dog in the domicile | 4 | Observational | Not serious^a b^ | Serious^f^ | Not serious^i^ | Serious^k^ | Not assessed^n^ | ⊕◯◯◯ VERY LOW | Absence of factors that increase the quality of evidence |
| Presence of chickens/other fowl at the domicile | 15 | Observational | Not serious^a b^ | Not serious^g^ | Not serious^i^ | Serious^k^ | Not serious^l^ | ⊕◯◯◯ VERY LOW | Absence of factors that increase the quality of evidence |
| Presence of domestic/farm/wild animals |  | | | | | | | | |
|  | Cattle: 4 | Observational | Serious^a d^ | Not serious^e^ | Not serious^i^ | Serious^k^ | Not assessed^n^ | ⊕◯◯◯ VERY LOW | Absence of factors that increase the quality of evidence |
|  | Cat: 5 | Observational | Serious^a d^ | Not serious^e^ | Not serious^i^ | Serious^k^ | Not assessed^n^ | ⊕◯◯◯ VERY LOW | Absence of factors that increase the quality of evidence |
|  | Pig: 6 | Observational | Not serious^a b^ | Not serious^e^ | Not serious^i^ | Serious^k^ | Not assessed^n^ | ⊕◯◯◯ VERY LOW | Absence of factors that increase the quality of evidence |
| Prior contact with infected household member, relatives or neighbors |  |  |  |  |  |  |  | ⊕⊕◯◯  LOW | Evidence raised to a level considering the observed effect measures in the set of variables |
|  | Relatives: 9 | Observational | Serious^a d^ | Serious^f^ | Not serious^i^ | Not serious^j^ | Not assessed^n^ | ⊕◯◯◯ VERY LOW | Absence of factors that increase the quality of evidence |
|  | Neighbors: 4 | Observational | Serious^a d^ | Not serious^e^ | Not serious^i^ | Not serious^j^ | Not assessed^n^ | ⊕◯◯◯ VERY LOW | Absence of factors that increase the quality of evidence |
| Malnutrition | 5 | Observational | Serious^a d^ | Serious^f^ | Not serious^i^ | Serious^k^ | Not assessed^n^ | ⊕◯◯◯ VERY LOW | Absence of factors that increase the quality of evidence |
| Accessible backyard at the domicile or nearby | 7 | Observational | Not serious^a b^ | Not serious^e^ | Not serious^i^ | Not serious^j^ | Not assessed^n^ | ⊕⊕◯◯ LOW | Absence of factors that increase the quality of evidence |
| Socioeconomic/living conditions |  |  |  |  |  |  |  | ⊕⊕  LOW | Evidence raised to a level considering the observed effect measures in the set of variables |
|  | Water treatment: 6 | Observational | Not serious^a b^ | Serious^f^ | Not serious^i^ | Serious^k^ | Not assessed^n^ | ⊕◯◯◯ VERY LOW | Absence of factors that increase the quality of evidence |
|  | Sewage network: 6 | Observational | Not serious^a b^ | Serious^f^ | Not serious^i^ | Serious^k^ | Not assessed^n^ | ⊕◯◯◯ VERY LOW | Absence of factors that increase the quality of evidence |
|  | Garbage collection: 6 | Observational | Not serious^a b^ | Serious^f^ | Not serious^i^ | Serious^k^ | Not assessed^n^ | ⊕◯◯◯ VERY LOW | Absence of factors that increase the quality of evidence |
|  | Suitable flooring: 8 | Observational | Serious^a d^ | Serious^h^ | Not serious^i^ | Serious^k^ | Not assessed^n^ | ⊕◯◯◯ VERY LOW | Absence of factors that increase the quality of evidence |
|  | Home finishing/ Roof conditions: 14 | Observational | Serious^a d^ | Serious^h^ | Not serious^i^ | Serious^k^ | Not serious^l^ | ⊕◯◯◯ VERY LOW | Absence of factors that increase the quality of evidence |

^a^ Observational studies (level of evidence starts at low)

^b^ Studies with better methodological quality and greater weight did not present significant risks of additional bias (beyond those inherent to observational studies)

^c^ Most studies with high risk of additional bias (beyond those inherent to observational studies)

^d^ Studies with greater weight presented significant risks of additional bias (beyond those inherent to observational studies)

^e^ Non-significant heterogeneity

^f^ Significant heterogeneity

^g^ High heterogeneity, but explained by sensitivity analyses

^h^ High heterogeneity not explained by sensitivity analyses

^i^ Absence of significant indirect evidence

^j^ Low imprecision

^k^ High imprecision

^l^ Absence of significant publication bias

^m^ Presence of significant publication bias

^n^ Not assessed due to lack of sufficient publications

^o^ Considering the entire context of socioeconomic variables
